# The role of distinct *APOBEC/ADAR* mRNA levels in mutational signatures linked to aging and ultraviolet radiation

**DOI:** 10.1038/s41598-024-64986-6

**Published:** 2024-07-04

**Authors:** Ahmadreza Niavarani

**Affiliations:** Digestive Oncology Research Center, Digestive Disease Research Institute, Tehran University of Medical Sciences, Tehran, Iran

**Keywords:** *APOBEC/ADAR* mRNA levels, *APOBEC/ADAR* mutation, Cancer mutation, Mutational signature, Cancer genomics, Cancer genetics, Genome, Mutation

## Abstract

The APOBEC/AID family is known for its mutator activity, and recent evidence also supports the potential impact of ADARs. Furthermore, the mutator impacts of *APOBEC/ADAR* mutations have not yet been investigated. Assessment of pancancer TCGA exomes identified enriched somatic variants among exomes with nonsynonymous *APOBEC1, APOBEC3B, APOBEC3C*, *ADAR,* and *ADARB1* mutations, compared to exomes with synonymous ones. Principal component (PC) analysis reduced the number of potential players to eight in cancer exomes/genomes, and to five in cancer types. Multivariate regression analysis was used to assess the impact of the PCs on each COSMIC mutational signature among pancancer exomes/genomes and particular cancers, identifying several novel links, including SBS17b, SBS18, and ID7 mainly determined by *APOBEC1* mRNA levels; SBS40, ID1, and ID2 by age; SBS3 and SBS16 by *APOBEC3A/APOBEC3B* mRNA levels; ID5 and DBS9 by DNA repair/replication (DRR) defects; and SBS7a-d, SBS38, ID4, ID8, ID13, and DBS1 by ultraviolet (UV) radiation/*ADARB1* mRNA levels. *APOBEC/ADAR* mutations appeared to potentiate the impact of DRR defects on several mutational signatures, and some factors seemed to inversely affect certain signatures. These findings potentially implicate certain *APOBEC/ADAR* mutations/mRNA levels in distinct mutational signatures, particularly *APOBEC1* mRNA levels in aging-related signatures and *ADARB1* mRNA levels in UV radiation-related signatures.

## Introduction

The APOBEC/AID (apolipoprotein B mRNA editing enzyme, catalytic polypeptide/activation-induced cytidine) family of cytidine deaminases, which are involved in a wide range of physiological and developmental activities, whether in DNA (reviewed in^1^) or RNA^2,3^, have also been implicated in mutagenesis across various cancers^4–7^. Two single base substitution mutational signatures with predominant C > T (SBS2) and C > G (SBS13) variants have been attributed to the APOBEC/AID family^5,7^, in addition to potentially one double base substitution signature with predominant CC > NN (DBS11) variants^8^. While both APOBEC3B (A3B)^6,9^ and APOBEC3A (A3A)^9,10^ are strongly linked to APOBEC mutational signatures, the implication of other family members with nuclear distribution, including APOBEC1 (A1)^11^, APOBEC3C (A3C)^12^, APOBEC3F (A3F)^13^, APOBEC3H (A3H)^14^, and AID^15^, cannot be excluded. AID has long been implicated in B-cell malignancies^16,17^. Although much attention has been given to the high mRNA levels of *APOBEC/AICDA* genes^6,18^ and their copy number^9^ or single nucleotide variations^19^, these might not fully explain all APOBEC mutagenic impacts observed in cancer genomes. For instance, Kanke et al. reported some tumors of the breast, ovary, and uterus with a predominant SBS2, which did not show high *APOBEC* mRNA levels^20^. It seems that the implication of other factors, including *APOBEC* somatic gene mutations, has been simply overlooked. On the other hand, there is evidence showing that known adenosine deaminases acting on RNA (ADARs) may also act on DNA. These include the DNA mutator activity of ADAR1 in *MYCC* and a class switch recombination region (Ig-Sμ)^21^; deamination of adenosine at dA-C mismatches of the DNA‒RNA hybrids by both ADAR1 and ADAR2^22^; and some indirect evidence^23,24^. However, the role of *APOBEC/ADAR* mutations has not yet been clarified in cancer. Our pancancer analysis shows that these genes are themselves subject to somatic mutations, although at a low frequency.

A preliminary analysis showed that pancancer exomes with any of the fourteen *APOBEC/ADAR* genes mutated showed significantly more single nucleotide variants (SNVs) and insertions/deletions (indels). Since this could suggest either a “driver” or a “passenger” role for *APOBEC/ADAR* mutations in cancer hypermutation, the burden of genomic variants was assessed in exomes with nonsynonymous versus synonymous *APOBEC/ADAR* mutations to check their true driver impact, showing that mutations in *A1, A3B, A3C*, and *ADAR* are correlated with indel variants, while *ADARB1* mutations are correlated with certain SNVs. Follow-up analyses estimated the roles of *APOBEC/ADAR* mutations and mRNA levels in mutational signatures among various cancer types, as adjusted for defects in DNA repair/replication (DRR) pathways.

## Results

Assessment of 10,295 tumor exomes identified 3,600,963 unique somatic variants, of which 3,427,680 (95.2%) were found to be SNVs and 173,109 (4.8%) were of the indel type. Approximately 2,710,172 (75.3%) of the somatic variants were found to be nonsynonymous variants occurring within coding regions, and the remaining 24.7% (890,791) were either intronic or synonymous exonic, herein called synonymous mutations. Collectively, 874 exomes (8.5%) showed at least one nonsynonymous somatic mutation in one or more *APOBEC/ADAR* genes, including *APOBEC1, APOBEC2, APOBEC3A, APOBEC3B, APOBEC3C, APOBEC3D, APOBEC3F, APOBEC3G, APOBEC3H, APOBEC4*, *AICDA, ADAR, ADARB1,* and *ADARB2*, with 188 exomes (21.5%) showing more than one mutated gene. The mean number of total *APOBEC/ADA*R mutations varied between 0 per exome in PCPG, TGCT, THYM, and UVM to 0.36 and 0.62 in SKCM and UCEC, respectively (Supplementary Table S1). The most common nonsynonymous mutations were seen in *ADARB2* (198; 1.92% per exome), whereas *A3A* (40; 0.39% per exome) was found to be the least mutated. In general, the *ADAR* family tended to have a higher mean number of mutations (178; 1.73% per exome) than the *APOBEC* family (68; 0.66% per exome). None of the nonsynonymous *APOBEC/ADAR* mutations reported by the TCGA-MC3 catalog were annotated as having a *low Impact*, 68 (5.3%) were classified as having a *modifier Impact*, and 1,204 (94.7%) were classified as having a *moderate/high Impact*. Similarly, none of the nonsynonymous DRR mutations were annotated as having a *low Impact*, 562 (2.8%) were classified as having a *modifier Impact*, and 19,292 (97.2%) were classified as having a *moderate/high Impact*.

### Essentially, all APOBEC/ADAR-mutated exomes showed a higher number of somatic variants

The mean SNV burden was found to be enriched in those exomes with any *APOBEC/ADAR* mutations compared to those with wild-type ones, varying between 13.9-fold for *ADARB2*-mutated exomes to 25.5-fold for *A2*-mutated ones (Supplementary Table S2). Likewise, the mean indel burden was enriched in essentially all *APOBEC/ADAR*-mutated exomes compared to their wild-type counterparts, varying between 5.9-fold for *AICDA-*mutated exomes and 11.9-fold for *A1*-mutated ones.

### T > N SNVs were correlated with ADARB1 mutations, as were certain indels with A1, A3B, A3C, and ADAR mutations

In a more stringent assessment, the mean numbers of genomic T > N, C > N, Ind-T, and Ind-C variants were compared in exomes with nonsynonymous versus synonymous *APOBEC/ADAR* mutations. Assessment of the SNVs showed that the mean number of T > N variants was enriched 2.8-fold in nonsynonymous *ADARB1* mutant exomes (Supplementary Table S3). On the other hand, the mean number of Ind-T variants was enriched 6.0-fold in nonsynonymous *A1* mutant exomes, 5.0-fold in nonsynonymous *A3B* mutants, 22.2 times higher in nonsynonymous *A3C* mutants, and 2.3-fold in nonsynonymous *ADAR* mutants, and the mean number of Ind-C variants was 17.2-fold in nonsynonymous *A3C* mutant exomes and 2.1-fold in nonsynonymous *ADAR* mutant exomes (Supplementary Table S3).

### Assessing the impacts of APOBEC/ADAR mutations and mRNA levels on *cancer* mutational signatures

**Figure 1:**
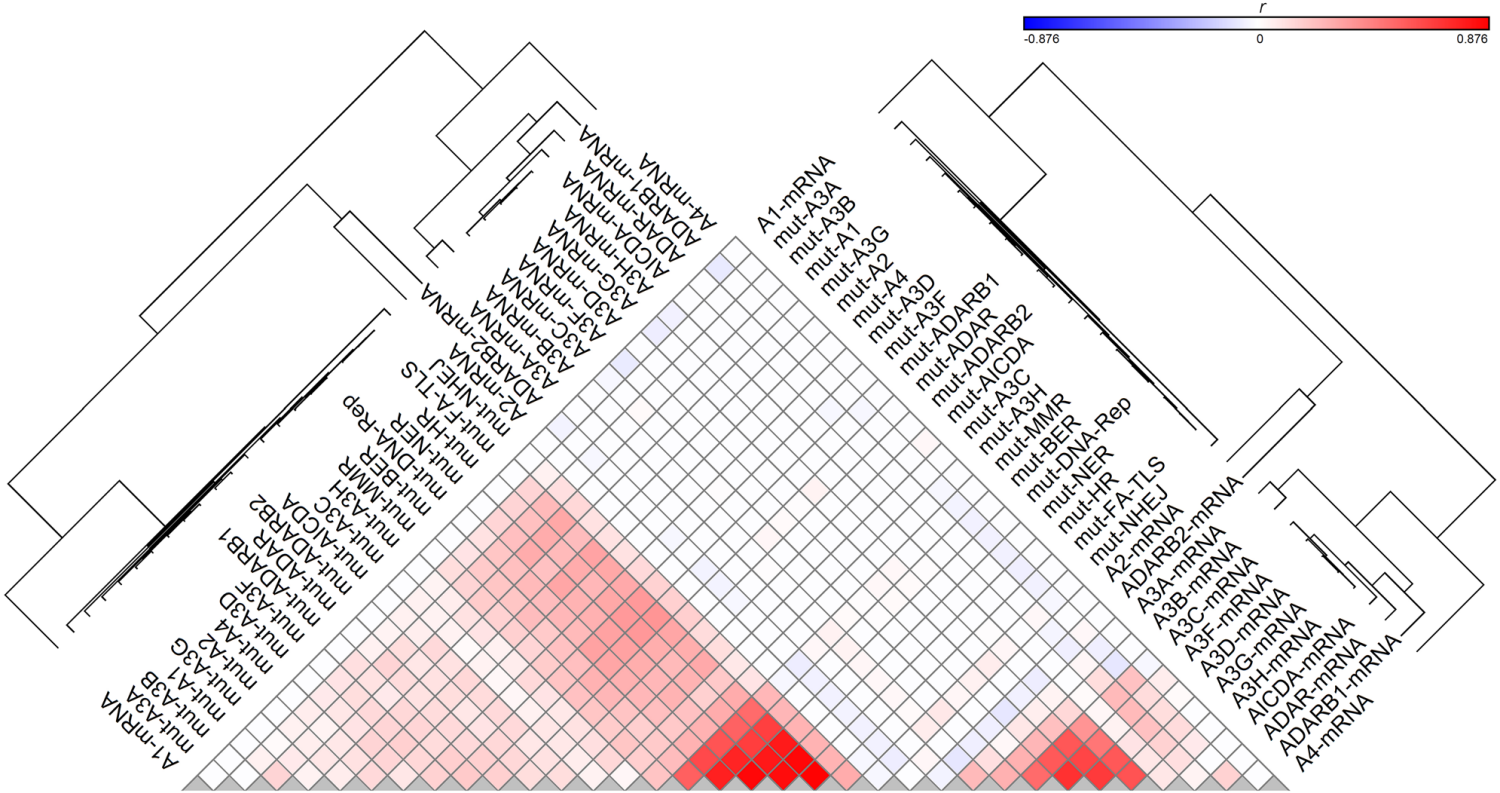
Clustering analysis of the correlation *(r)* between potential covariates implicated in cancer mutagenesis, including *APOBEC/ADAR* mutations and mRNA levels as well as DNA repair/replication (DRR) defects. Pearson regression analysis was used in order to estimate the correlations. DRR defects were very closely clustered with each other, followed by *APOBEC/ADAR* mutations. On the other hand, *A3A* and *A3B* mRNA levels were closely clustered to each other, and *A3C, A3D, A3F, A3G,* and *A3H* mRNA levels were also closely co-clustered.

**Figure 2:**
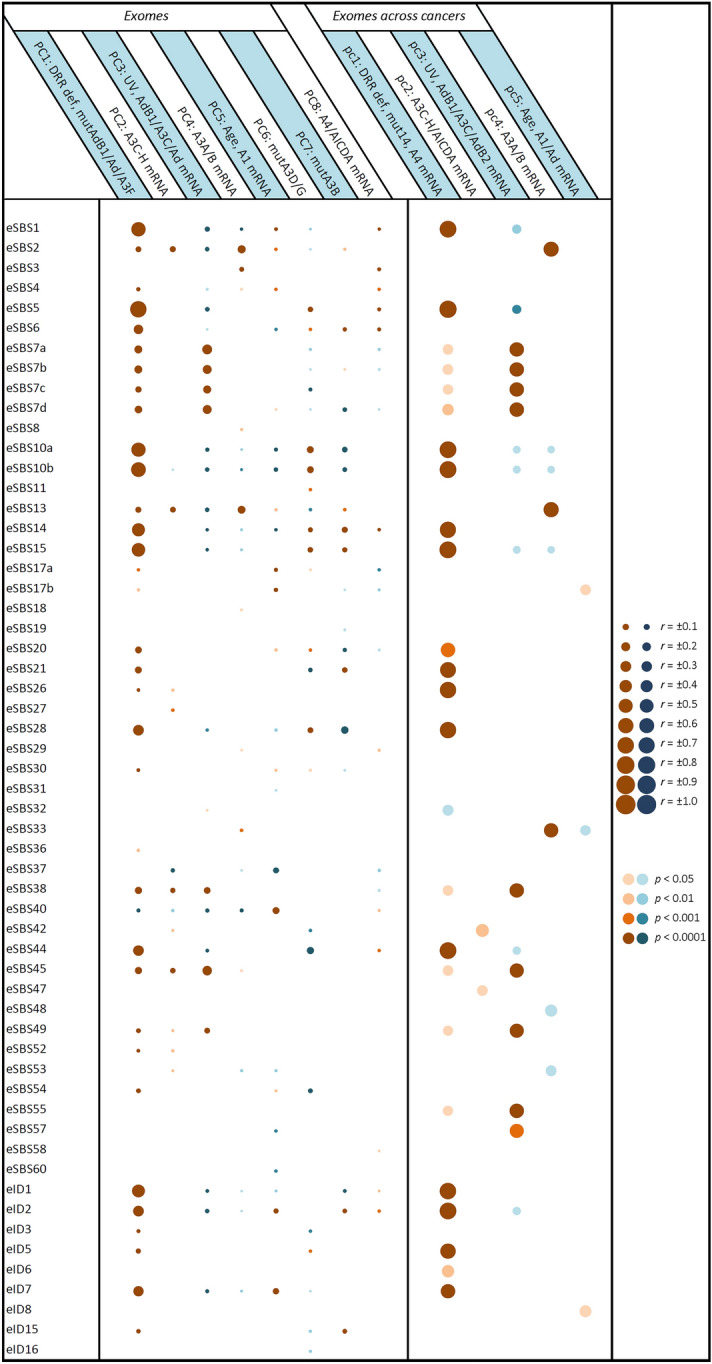
Correlation of pancancer exome mutational signatures with principal components potentially implicated in cancer mutagenesis. Multivariate forward linear regression analysis was performed using eight/five varying PCs. Left; correlation across exome samples regardless of the cancer types. Right; correlation of mean exome values across 33 cancers. Brown and blue bubbles indicate positive and negative impacts, respectively, with darker shadows indicative of more significant correlations. The main determinant PCs were at least partially concordant among exomes and cancers in the following mutational signatures: eSBS1, eSBS2, eSBS5, eSBS7a-d, eSBS10a,b, eSBS13, eSBS14, eSBS15, eSBS17b, eSBS20, eSBS21, eSBS26, eSBS28, eSBS33, eSBS38, eSBS44, eSBS45, eSBS49, eID1, eID2, eID5, and eID7. A1, *APOBEC1*; A2, *APOBEC2*; A3, *APOBEC3*; A4, *APOBEC4*; Ad, *ADAR*; AdB1, *ADARB1*; AdB2, *ADARB2*; mut14, mutation of fourteen *APOBEC/ADAR* genes. The “e” prefix denotes the exome signatures.

**Figure 3:**
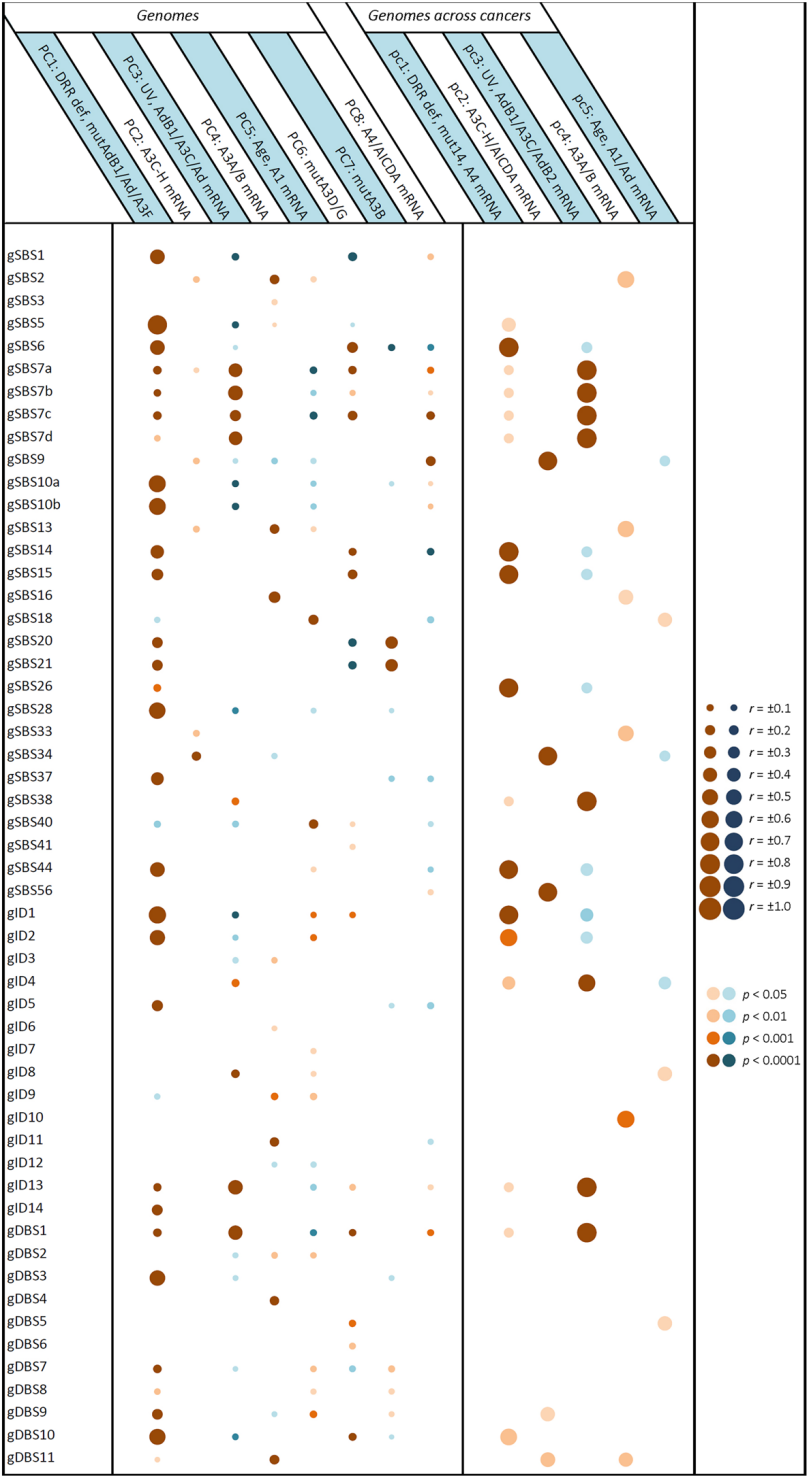
Correlation of pancancer genome mutational signatures with principal components potentially implicated in cancer mutagenesis. Multivariate forward linear regression analysis was performed using eight/five varying PCs. Left; correlation across genome samples regardless of the cancer types. Right; correlation of mean genome values across 33 cancers. Brown and blue bubbles indicate positive and negative impacts, respectively, with darker shadows indicative of more significant correlations. The main determinant PCs were at least partially concordant among genomes and cancers in the following mutational signatures: gSBS2, gSBS5, gSBS6, gSBS7a-d, gSBS9, gSBS13, gSBS14, gSBS15, gSBS16, gSBS18, gSBS26, gSBS34, gSBS38, gSBS44, gSBS56, gID1, gID2, gID4, gID8, gID13, gDBS1, gDBS10, and gDBS11. A1, *APOBEC1*; A2, *APOBEC2*; A3, *APOBEC3*; A4, *APOBEC4*; Ad, *ADAR*; AdB1, *ADARB1*; AdB2, *ADARB2*; mut14, mutation of fourteen APOBEC/ADAR genes. The “g” prefix denotes the genome signatures.

**Figure 4:**
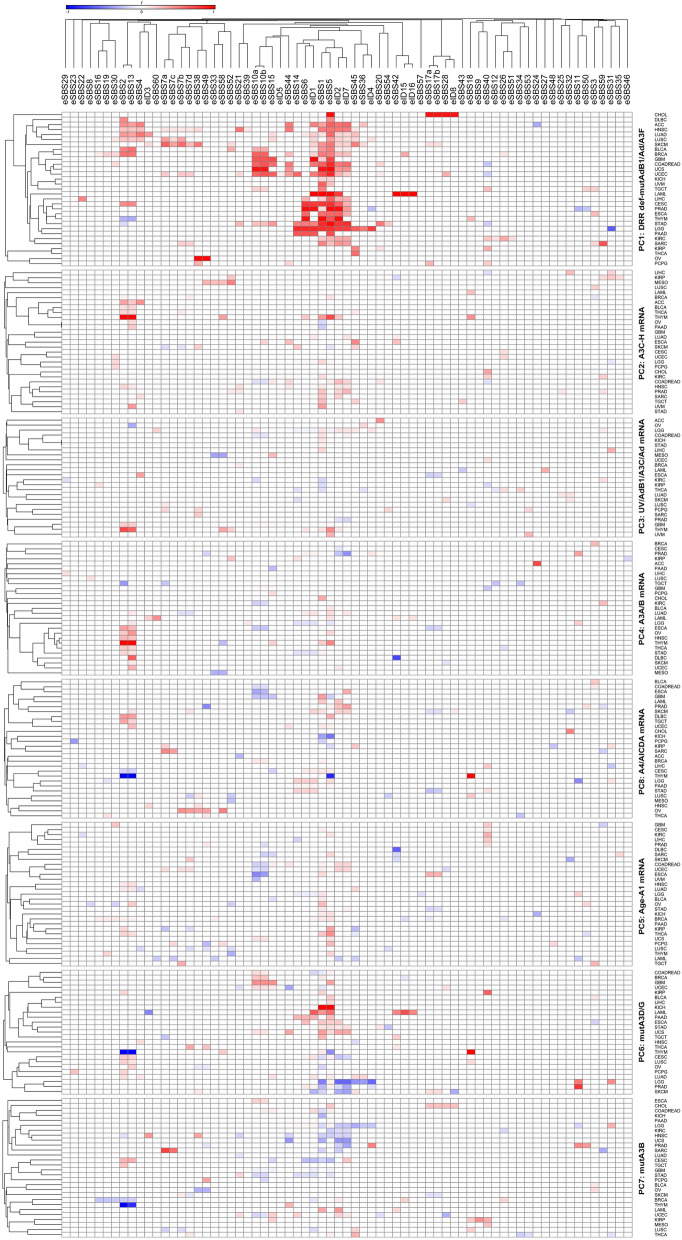
Detailed correlation of the exome signatures with principal components (PCs) potentially implicated in cancer mutagenesis in particular cancers, as stratified by PCs and clustered using Pearson regression *r* coefficient. The PCs that positively determine the mutational signatures in at least net 10% of cancers (four or more) included PC1 (DRR defects-mut*AdB1/Ad/A3F*) correlated with eSBS1, eSBS2, eSBS4, eSBS5, eSBS7a,b,d, eSBS10a,b, eSBS13, eSBS14, eSBS15, eSBS20, eSBS33, eSBS38, eSBS40, eSBS44, eSBS45, eID1, eID2, and eID7; PC2 (*A3C-H* mRNA level) correlated with eSBS1, eSBS2, eSBS13, eSBS45, eID2, and eID7; PC4 (*A3A/B* mRNA level) correlated with eSBS2, eSBS5, and eSBS13; PC5 (Age-*A1* mRNA level) correlated with eSBS1, eSBS2, eSBS5, eSBS13, and eSBS40; PC6 (mut*A3D/G*) correlated with eSBS2, eSBS5, eSBS6, and eSBS10a,b; PC7 (mut*A3B*) correlated with eSBS5; and PC8 (*A4/AICDA* mRNA level) correlated with eSBS1 and eSBS6. A1, *APOBEC1*; A3, *APOBEC3*; Ad, *ADAR*; AdB1, *ADARB1*.

**Figure 5:**
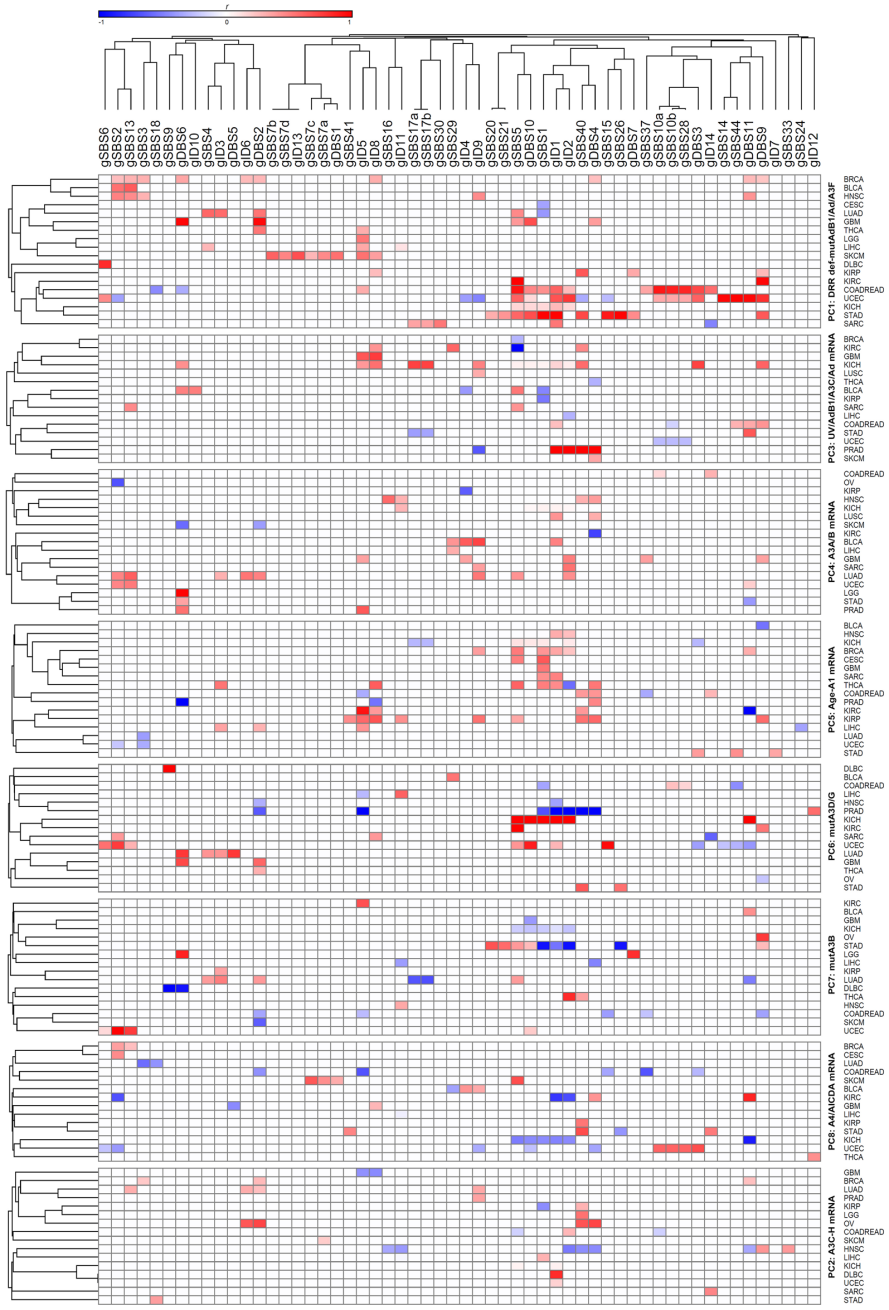
Detailed correlation of the genome signatures with principal components (PCs) potentially implicated in cancer mutagenesis in particular cancers, as stratified by PCs and clustered using Pearson regression *r* coefficient. The PCs that positively determine the mutational signatures in at least net 10% of cancers (three or more) included PC1 (DRR defects-mut*AdB1/Ad/A3F*) correlated with gSBS1, gSBS2, gSBS5, gSBS13, gID1, gID2, gID5, gID8, gDBS2, gDBS9, gDBS10, and gDBS11; PC2 (*A3C-H* mRNA level) correlated with gID1 and gDBS2; PC3 (UV-*AdB1/A3C/Ad* mRNA level) correlated with gSBS40 and gID8; PC4 (*A3A/B* mRNA level) correlated with gID1, gID2, gID9, and gDBS6; PC5 (age-*A1* mRNA level) correlated with gSBS1, gSBS5, gSBS40, gID1, gID2, gID5, gID8, and gDBS4; PC6 (mut*A3D/G*) correlated with gSBS5; and PC7 (mut*A3B*) correlated with gSBS2. A1, *APOBEC1*; A3, *APOBEC3*; Ad, *ADAR*; AdB1, *ADARB1*.

**Figure 6:**
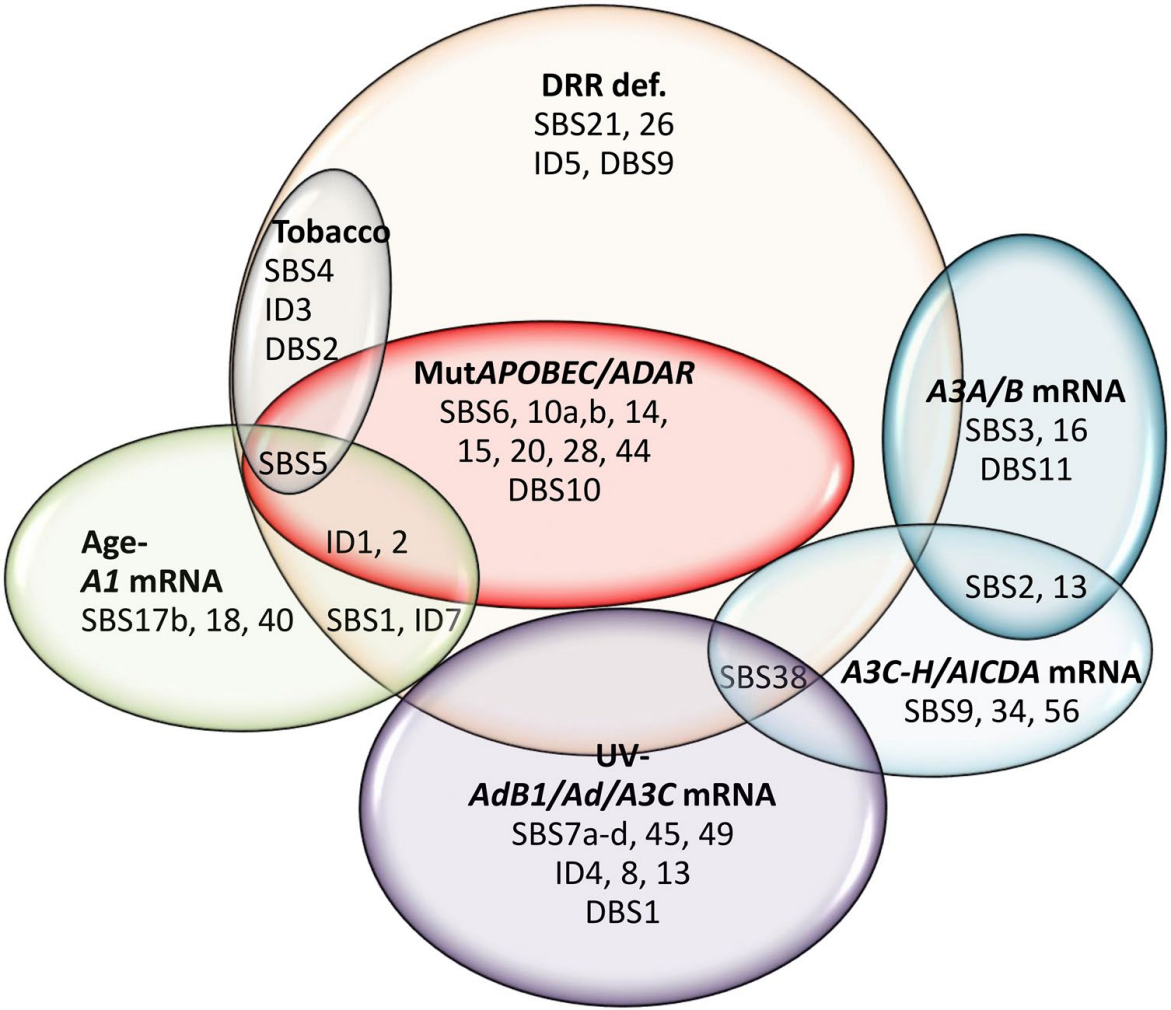
Collective clustering of mutational signatures linked to different factors across various cancers. Those mutational signatures that were found to be correlated with a particular factor in at least two analyses (Figs. 2, 3, 4, 5) were considered to belong to that cluster.

Regression followed by clustering analysis was performed using 10,126 samples with WES- and/or WGS-identified mutational signatures, identifying that many of the potential endogenous factors closely co-clustered with each other, including all DRR defects and *APOBEC/ADAR* mutations; *A3A* and *A3B* mRNA levels co-clustered with each other; and *A3C, A3D, A3F, A3G,* and *A3H* mRNA levels co-clustered with each other (Fig. 1). Hence, it was next attempted to reduce the number of potentially implicated covariates using principal component (PC) analysis. Assessment of 37 potential covariates, including age and UV factor, along with *APOBEC/ADAR* factors and DRR defects, reduced the number of potential covariates to eight PCs (explaining 49.5% of the variability) in pancancer exome (9,493 samples)/genome (773 samples) analyses (Supplementary Table S4). These included (1) seven DRR defects and mutations in *ADARB1, ADAR,* and *A3F;* (2) *A3C, A3D, A3F, A3G,* and *A3H (A3C-H)* mRNA levels; (3) UV factor and *ADARB1/A3C/ADAR* mRNA levels; (4) *A3A* and *A3B* mRNA levels; (5) age and *A1* mRNA levels; (6) *A3D* and *A3G* mutations; (7) *A3B* mutation; and (8) *A4* and *AICDA* mRNA levels. A similar analysis of the mean exome/genome covariates among 33 cancer types resulted in five PCs (explaining 77.6% of the variability; Supplementary Table S5), including (1) seven DRR defects, all *APOBEC/ADAR* mutations, and *A4* mRNA levels; (2) *A3C-H* and *AICDA* mRNA levels; (3) UV factor and *ADARB1/A3C/ADARB2* mRNA levels; (4) *A3A* and *A3B* mRNA levels; and (5) age and *A1/ADAR* mRNA levels. Multivariate correlations of the pancancer exome and genome signatures with the aforementioned PCs are shown in Figs. 2 and 3, respectively, and clustering analyses of the positive correlations are illustrated in Supplementary Figure S1. Furthermore, the correlations of the exome and genome signatures with PCs across 33 particular cancers are depicted in Figs. 4 and 5, respectively, as well as Supplementary Figures S2 and S3, respectively. Collectively, these findings can be summarized below, as illustrated in Fig. 6.

### UV-ADARB1 mRNA cluster

Seven signatures are known to be UV radiation-related, which were also found to be correlated with PC3 representing UV factor and *ADARB1/A3C/ADAR* mRNA levels (Fig. 6; Supplementary Figure S4). These include SBS7a,b,c,d, SBS38, ID13, and DBS1. Furthermore, the unknown genome ID4 (gID4) and the so-called artifact exome signatures SBS45 (eSBS45) and SBS49 (eSBS49) were mainly determined by this component. Not surprisingly, these signatures were primarily associated with significant DRR defects as well. More detailed analysis showed that SBS7a-d, SBS38, ID13, and DBS1 were merely or mainly determined by UV factor, while ID4 was determined by only the *ADARB1* mRNA level, and ID8 was determined by both *ADARB1* and *ADAR* mRNA.

### A3A/B mRNA cluster

Pancancer analysis across both samples and cancer types identified both exome and genome signatures SBS2 and SBS13 highly determined by *A3A/A3B (A3A/B)* mRNA levels (Fig. 6; Supplementary Figure S5). These findings were consistent with analyses of the exome mutational signatures across particular cancers, showing that eSBS2 was determined by *A3A/B* mRNA (THYM, OV, ESCA, THCA, STAD, HNSC) as well as *A3C-H* mRNA (THYM, ACC, HNSC, THCA, BLCA, BRCA), age-*A1* mRNA (KIRP, THCA, HNSC, BRCA, LUAD), and DRR defects in various cancers (Fig. 4). eSBS13 was also determined by *A3A/B* mRNA (THYM, OV, DLBC, ESCA, THCA, STAD, HNSC, UCEC, SKCM, LGG, LUAD), *A3C-H* mRNA (THYM, HNSC, THCA, BLCA, PAAD, ACC, OV), age-*A1* mRNA (OV, THCA, HNSC, BRCA, LUAD), and DRR defects in various cancers. However, the genome signatures SBS2 (gSBS2) and SBS13 (gSBS13) were found to be determined solely by DRR defects in particular cancers (Fig. 5). As expected, gDBS11 was also determined by the *A3A/B* mRNA level. Furthermore, HR-related eSBS3 and alcohol-related gSBS16 were found to be highly determined by *A3A/B* mRNA levels.

### Age-APOBEC1 (A1) mRNA cluster

The so-called 5FU-related eSBS17b and the ROS-related gSBS18 were found to be determined by age-*A1* mRNA level, as did the unknown SBS40 (Fig. 6; Supplementary Figure S6). Assessment of individual cancers (CESC, GBM, KIRC, LIHC, PRAD) across exomes; KIRP, KIRC, COADREAD across genomes) corroborated the correlation of SBS40 with age-*A1* mRNA level. A combined impact of the DRR-*ADARB1/ADAR/A3F* mutations and age-*A1* mRNA levels (BLCA, BRCA, CESC, COADREAD, ESCA, KICH, LGG, OV, STAD, THCA, UCEC across exomes; BRCA, CESC, GBM, KICH, SARC, THCA across genomes) was seen in aging-related SBS1. Likewise, the combined impact of the DRR-*ADARB1/ADAR/A3F* mutations and age-*A1* mRNA levels was also seen for indel signatures ID1, ID2, and ID7 in pancancer analyses as well as in certain cancers. A more detailed analysis of PC1 covariates identified SBS40, ID1, and ID2 to be determined mainly by age, while SBS17b, SBS18, and ID7 were determined mainly by *A1* mRNA level.

### Defective DRR cluster

This constituted the largest cluster by far, contributing to many mutational signatures. In addition to SBS21 and SBS26, which are currently attributed to some MMR defects, both unknown ID5 and DBS9 were also determined by DRR defects across exomes and/or genomes (Fig. 6). One subcluster consisted of signatures that were also determined by tobacco exposure, including the known signatures SBS4, ID3, and DBS2, as well as the so-called aging-related SBS5. Another subcluster consisted of SBS6, SBS10a,b, SBS14, SBS15, SBS20, SBS28, SBS44, ID1, ID2, and DBS10, which are currently known or predicted to be related to DRR defects, but they were also found to be determined by *APOBEC/ADAR* mutations (Fig. 6; Supplementary Figure S7). As discussed earlier, the mutational signatures SBS1, ID1, ID2, and ID7 were also determined by age-*A1* mRNA levels. Intriguingly, mut*A3B* (PC7) was also seen to inversely impact several mutational signatures across various cancers, particularly SBS1, SBS5, ID1, ID2, and ID7 (Fig. 4). Likewise, an apparently inverse impact was seen for other covariates in certain cancers, particularly those of mut*A3D/G* (most commonly affecting SBS1, SBS44, and ID2) and UV-*ADARB1/A3C/ADAR* mRNA levels (most commonly affecting SBS10a,b and SBS28).

### A3C-A3H/AICDA mRNA cluster

Although the variations in *A3C, A3D, A3F, A3G,* and *A3H (A3C-H)* and *AICDA* mRNA levels tended to be separate across particular exomes/genomes, they were very close across different cancer types. In light of this, three mutational signatures, including *PolH*-related SBS9, unknown SBS34, and the so-called artifact SBS56, were found to be determined by *A3C-H/AICDA* mRNA levels across both genomes and cancer types (Fig. 6). As mentioned earlier, both SBS2 and SBS13 were also determined by *A3C-H* mRNA levels in certain cancers.

## Discussion

In this study, DRR defects were found to be a major determinant of various mutational signatures, sometimes even for those primarily known to be related to other factors such as aging, *A3A/B* mRNA levels, or UV exposure. Since the high mRNA levels of *A1, A3A/B,* and *ADARB1* were all found to determine distinct mutational signatures in both wild-type and mutated DRR subgroups, it is reasonable to believe that these factors can act independently of the DRR defects as well. This is not surprising, considering the independent regulation of the expression levels of the aforementioned genes. However, this was not the case for the *APOBEC/ADAR* mutations, which almost always occurred in the presence of a DRR defect. Therefore, it can be concluded that while the *APOBEC/ADAR* mutations have arisen from a DRR defect, they potentiate the impact of the latter on specific mutational signatures. Several mutational signatures were found to be solely determined by DRR defects, including SBS21, SBS26, ID5, and DBS9. Boichard et al. showed that mutations in the *A1, A4, AICDA,* and *A3* subfamily predicted the mutational burden regardless of MMR or *POLD1/POLE* defects, with no assessment of individual APOBEC mutations or other DRR defects^25^. Our preliminary analysis showed that rather infrequent *APOBEC/ADAR* mutations occur more commonly in hypermutated exomes. Since synonymous mutations are supposed to be of no functional impact and occur rather constantly over time^26^, in this study the burden of genomic variants was assessed among those exomes with nonsynonymous *APOBEC/ADAR* mutations compared to those with synonymous ones, showing that T > N SNVs were enriched in *ADARB1*-mutant exomes, while all indels were found to be enriched in *A3C* and *ADAR*-mutant exomes and Ind-T variants were enriched in *A1* and *A3B*-mutant exomes.

The high number of potentially implicated covariates made a PC analysis inevitable, although complicating the attribution of distinct mutational signatures to individual *APOBEC/ADAR* aberrations, particularly infrequent *APOBEC/ADAR* mutations. PC analysis reduced the number of potentially implicated factors, and multivariate models using the measured principal components proposed novel correlations for the so-called artifact/unknown mutational signatures, including SBS34 and SBS56 (determined by *A3C-H/AICDA* mRNA level), SBS40 (determined by age), ID5 and DBS9 (determined by DRR defects), and SBS45, SBS49, and ID4 (determined by UV-*ADARB1/A3C/ADAR* mRNA level). SBS34 is commonly seen among DLBC, STAD, and PAAD and shows asymmetry toward the intergenic regions, as does SBS9^27^. SBS40 has been proposed to be related to environmental factors because of its accumulation with age in some cancer types^28^. SBS45 and SBS49 are claimed to be possible sequencing artifacts, the former due to 8-oxo-guanines introduced during sequencing^27^. SBS56 has been reported to be a marker of AKT inhibitor sensitivity in some cancer cell lines^29^, further undermining an artifact nature.

Moreover, some findings were apparently discrepant from the current literature, including SBS3, SBS16, SBS17b, SBS18, ID4, and ID8. SBS16 has been suggested to be alcohol-related in ESCC^30^, while it was found here to be determined by *A3A/A3B* expression levels. Whether this discrepancy is due to the genomic hypomethylating impact of ethanol, which increases *A3C-H* mRNA levels^31^, or because of a shift in A3A function from so-called physiological 5hmC demethylation to potentially oncogenic C demethylation^32^ needs to be investigated in more detail. The so-called 5FU-related SBS17b^33^ and ROS-related SBS18^27^ were also found to be determined by *A1* mRNA levels. In addition, the clock-like signature SBS1^34^ was found to be determined by DRR defects and *A1* mRNA levels, while the other clock-like signature SBS5^34^, which has also been reported to be smoking-related^35^, was determined by DRR defects, tobacco use, *APOBEC/ADAR* mutations, and age. Intriguingly, at least three AID/APOBEC proteins, including AID, A3A, and A3B, have been reported to efficiently deaminate dC neighboring DNA damage induced by oxidation or alkylation^36^, a function that might be implicated in this case as well. *A1* mRNA levels have already been linked to cancer indels, in particular Ind-T ones^18^, and it was shown here that at least ID2 (Del-T) and ID7 (Del-C/T) signatures were determined by *A1* mRNA levels in addition to the known DRR defect impact^37,38^, further characterizing the *A1* mRNA impact on cancer genomes. The impacts of age on ID1 (Ins-T) and ID8 (long Del) also seem to be mediated through *A1* mRNA. Unknown ID4, which has been suggested to be TOP1-related^39^, was determined by *ADARB1* mRNA level, and the so-called radiation-related ID8 was determined by *ADARB1* and *ADAR* mRNA levels across genomes. Recent studies have shown that ADAR1 (encoded by *ADAR*) can edit DNA:RNA hybrids that form during transcription and DNA replication^40,41^. Specifically, ADAR2 (encoded by *ADARB1*) has been reported to play a role in editing such hybrids needed for dsDNA break repair and genomic stability^40^. This is the function which might be implicated in indel mutational signatures that are supposed to be UV-related, including ID4, ID8, and ID13. Since both AID and A3A have also been proposed to mediate skin cancer through chronic inflammation and mutational events, respectively^42,43^, more detailed studies might be warranted to explore the true endogenous UV mediator. The supposedly HR-related SBS3 was determined by *A3A/B* mRNA levels. Whether this is a real link or *A3A/B* mRNA is a proxy for other factors (Fig. 1) needs to be determined by further investigation.

The expression of *APOBEC3* family members is regulated by different factors, including B-cell receptor signaling, noncanonical NF-κB signaling, and SF3B1 inhibition^44,45^. Additionally, the expression of *APOBEC3* genes can be regulated by epigenetic modifications, such as DNA methylation and histone acetylation^46^. However, there is much evidence supporting some links between *APOBEC/ADAR* activation and environmental mutagens, including viruses, tobacco, and UV radiation. Not unexpectedly, HPV-positive HNSCs show higher mRNA levels of *A3A, A3B*, and *A3H* than HPV-negative HNSCs^47^. It has been reported that increased expression of *A3B* in response to ionizing radiation could contribute to the acquisition of radiation resistance in cancer cells^48^, and radiotherapy is followed by APOBEC mutagenesis^49^. A3G has also been shown to be activated by UV radiation and rescue cells from its detrimental DNA effects^50,51^, as well as enhancing double-strand break (DSB) repair in leukemia and lymphoma cells^16^, making them radioresistant. Likewise, assessment of tobacco-associated cancers suggested that the cellular machinery underlying APOBEC signatures was activated by tobacco smoke^35^, and APOBEC rather than tobacco-associated mutagenesis predominated in two series of bladder cancers^52^. On the other hand, smoking has been shown to repress *ADAR* expression, enhancing intracellular oxidative stress^53,54^.

One biologically plausible explanation for the inverse impacts of certain factors might be the competitive actions of homologous proteins, including those of mut*A3B* on *A1* mRNA-determined signatures SBS1, SBS5, ID1, ID2, and ID7. Certain *APOBEC/ADAR* proteins have been known to modulate each other through heterodimerization or coexpression. These include A2 dimerizing with and inhibiting A1^55^, ADAR1 (encoded by *ADAR*) dimerizing with and sequestering ADAR2 (encoded by *ADARB1*)^56^, and ADAR3 (encoded by *ADARB2*) downregulating ADAR2^57^. Likewise, gain- or loss-of-function variants of *APOBEC/ADAR* proteins have been reported, including an A3C variant (S188I) with increased dimerization of the protein and hypermutation of target sequences^58^ and an ADAR variant (P193A) destabilizing the protein-Z-DNA complex and affecting tumor cell proliferation^59^. Although ADAR3 has not been shown to have any catalytic activity thus far^60^, some SNVs in *ADARB2* show a consistent link to longevity across populations^61^, suggesting a functional role.

One limitation of the present study is that PC2 does not differentiate between the distinct roles of the co-regulated *A3C-H* and *AICDA* genes. Given the cellular location of the gene products, however, it is reasonable to believe that A3H and AID are the potential players among A3C-H/AID proteins. I also acknowledge that some proposed novel links might seem unexpected by currently known mechanisms. Whether or not the reciprocal nucleic acid changes described elsewhere^3^ are implicated in these novel links would be an intriguing field of future studies, including the unexpected T > C changes in SBS16 found to be linked to *A3A/B* mRNA levels. The fact that several highly significant associations were observed only in the much smaller genome rather than the exome subgroup (i.e., gSBS16, gSBS18, gSBS34, gSBS56, gID4, gID8, gID13, gDBS1, gSBS9, gDBS10, and gDBS11) might implicate some truly differential mutagenic mechanisms acting among intergenic versus genic regions.

In conclusion, this pancancer approach links several exome/genome mutational signatures with novel factors among APOBEC and ADAR families, including SBS17b, SBS18, and ID7 (*A1* mRNA level); SBS3 and SBS16 (*A3A/B* mRNA level); ID4 and ID8 (*ADARB1* mRNA level); SBS40, ID1, and ID2 (age); and ID5 and DBS9 (DRR defects). While some *APOBEC/ADAR* mutations potentiate the impact of DRR defects on certain mutational signatures, the impact of high expression levels of *A1, A3A/B, A3C-H/AICDA,* and *ADARB1* on distinct signatures can be independent of other mutagenic factors, while still modulating them. It is proposed that the mutagenic impacts of aging are at least partly mediated through the *A1* mRNA level, while the UV impacts are mainly mediated through *ADARB1* mRNA levels. The precise mechanisms that are involved need to be investigated in detail.

## Methods

### Patients and samples

The findings of the current study are based on data generated by the TCGA Research Network. A catalog of all somatic variants identified by whole-exome sequencing of 10,295 paired tumor-normal samples was obtained from sftp://tcgaftps.nci.nih.gov/tcgajamboree/mc3/pancan.merged.v0.2.7.PUBLIC.maf.gz, comprising a total of 3,600,963 variants from 33 tumor types. In brief, the MC3 version of the TCGA variants had been consistently called using seven methods with proven performance including MuTect (https://github.com/broadinstitute/mutect), Pindel (https://github.com/genome/pindel), Radia (https://github.com/aradenbaugh/radia), VarScan2 (http://dkoboldt.github.io/varscan/), SomaticSniper (https://github.com/genome/somatic-sniper), MuSE (https://github.com/danielfan/MuSE), and Indelocator (http://archive.broadinstitute.org/cancer/cga/indelocator), as described before^62^. Non-synonymous *APOBEC/ADAR* variants used for analysis included all variants but the silent, intronic, and flanking ones. The same rule was applied for the mutations in DRR pathway genes. The list of key DRR pathway genes was obtained from the Kyoto Encyclopedia of Genes and Genomes (KEGG)^63^, as follows: base excision repair (BER; hsa03410; 33 genes), nucleotide excision repair (NER) and transcription-coupled NER (hsa03420; 45 genes), mismatch repair (MMR; hsa03430; 22 genes), homologous recombination (HR; hsa03440; 41 genes), nonhomologous end-joining (NHEJ; hsa03450; 10 genes), and Fanconi anemia-translesion synthesis (FATLS; hsa03460; 54 genes), as well as DNA replication pathway (DNA-Rep; hsa03030; 35 genes), collectively 168 unique genes. All nonsynonymous exome variants were included for analysis of the potentially implicated pathways. A list of *APOBEC/AICDA/ADAR* gene mutations was extracted, in which mutations were classified into synonymous or nonsynonymous mutations, with the latter including missense, nonsense, splice site, and indel mutations.

The following open access (Level 3) databases were obtained from the Broad Institute GDAC Firehose (http://gdac.broadinstitute.org/): normalized *RNA-Seq* by Expectation–Maximization (RSEM) for genes (RSEM_genes_normalized_data, v.20160128) and clinical data, including vital status and days to death/last follow-up (Clinical_Pick_Tier1, v.20171224). The normalized RSEM data resulting from RNA sequencing were used as an estimate of TCGA gene expression profiles, and they were further normalized to *TATA-binding protein (TBP)* mRNA levels and then scaled to 1. Indels longer than one nucleotide were classified based on their 5’-end nucleotide. The absolute number of variants attributed to each mutational signature as detected by whole-exome or whole-genome sequencing (COSMIC release V89, May 2019) was obtained from Rosenthal’s study^62^ and used for assessment of the correlation with *APOBEC/ADAR* aberrations as well as DRR defects. This version includes 93 mutational signatures: SBS1-SBS60, ID1-ID17, and DBS1-DBS11. Three signatures including SBS7, SBS10, and SBS17 were represented by four, two, and two sub-signatures, respectively.

### Statistical analysis

Unpaired two-tailed Student’s t test (IBM SPSS, v.22) was used to compare the mean number of indel variants and SNVs among wild-type versus mutant APOBEC/ADAR exomes, as well as nonsynonymous versus synonymous *APOBEC/ADAR* mutant exomes, and the *Benjamini–Hochberg* false discovery rate (FDR) was used to adjust for multiple testing, with an acceptable FDR of up to 0.05. Principal component (PC) analysis was performed using the Varimax method with Kaiser Normalization (IBM SPSS, v.22) across 10,126 samples with WES (9,493)/WGS (773)-identified mutational signatures (140 having both) to reduce the number of 37 covariates, including 14 *APOBEC/ADAR* mutations, 14 *APOBEC/ADAR* mRNA levels, seven DRR pathway defects, age, and UV factor (positive in SKCM and UVM). An acceptable eigenvalue cutoff of 1.0 was used to select the PCs and a cutoff of 0.4 for significant rotated components. A similar PC analysis was performed using the mean exome/genome values of the aforementioned 37 covariates across 33 cancer types, with an eigenvalue cutoff of 1.5 used to select the PCs and a cutoff of 0.4 for significant rotated components. Next, forward linear regression analysis (IBM SPSS, v.22) was used to estimate the correlation of the mutational signatures with PCs (eight PCs for pancancer exome/genome analysis, and five for mean exome/genome values across 33 cancers), with an acceptable* P* value of up to 0.05. For mutational signatures that were found to be correlated with PC3 (UV-*ADARB1/A3C/ADAR* mRNA level) and PC5 (age*-A1* mRNA level), repeat detailed analysis was performed to identify the exact factor correlated with each signature. For those mutational signatures whose univariate Pearson regression analysis (IBM SPSS, v.22) suggested a potential link to tobacco use, this factor was also included in multivariate regression analysis. The third kind of multivariate regression analysis involving mutational signatures was performed using varying PCs (aforementioned eight) in particular cancers, including adrenocortical carcinoma (ACC; 91 exomes/0 genomes), bladder carcinoma (BLCA; 389/22), breast carcinoma (BRCA; 930/86), cervical/endocervical cancer (CESC; 271/20), cholangiocarcinoma (CHOL; 36/0), colon and rectal adenocarcinoma (COADREAD; 496/52), diffuse large B-cell lymphoma (DLBC; 31/6), esophageal carcinoma (ESCA; 182/0), glioblastoma multiforme (GBM; 362/35), head and neck squamous cell carcinoma (HNSC; 464/43), kidney chromophobe (KICH; 50/45), kidney clear cell carcinoma (KIRC; 334/27), kidney papillary cell carcinoma (KIRP; 251/33), acute myeloid leukemia (LAML; 139/0), low-grade glioma (LGG; 512/18), hepatocellular carcinoma (LIHC; 311/52), lung adenocarcinoma (LUAD; 528/37), lung squamous cell carcinoma (LUSC; 427/48), mesothelioma (MESO; 82/0), ovarian serous cystadenocarcinoma (OV; 385/27), pancreatic adenocarcinoma (PAAD; 174/0), pheochromocytoma/paraganglioma (PCPG; 182/0), prostate adenocarcinoma (PRAD; 484/19), sarcoma (SARC; 208/30), skin melanoma (SKCM; 412/37), stomach adenocarcinoma (STAD; 401/38), testicular germ cell tumor (TGCT; 149/0), thyroid carcinoma (THCA; 478/48), thymoma (THYM; 123/0), uterine corpus endometrial carcinoma (UCEC; 474/50), uterine carcinosarcoma (UCS; 57/0), and uveal melanoma (UVM; 80/0). Those correlations that were found in at least 2 out of 6 analyses (pancancer exomes/genomes, pancancer mean exomes/genome values across 33 cancers, and cancer-specific exomes//genomes) were considered to be consensus, and the data were used to cluster mutational signatures based on potential underlying factors. The median of the mean normalized *A3A* and *A3B* mRNA levels was used to classify *A3A/B* mRNA into low and high levels, and *APOBEC/ADAR* mutational status was considered to be positive when at least one of the corresponding genes was mutated. An unpaired two-tailed t test with Welch's correction (IBM SPSS, v.22) was used to compare the mean number of mutational signatures among paired subgroups, including wild type versus mutated DRR, wild type versus mutated *A3D/G*, wild type versus mutated *ADARB1/ADAR/A3F*, wild type versus mutated *APOBEC/ADAR,* low- versus high-*A1* mRNA level, low- versus high-*A3A/B* mRNA level, and low- versus high-*ADARB1* mRNA level. Gene-E (https://software.broadinstitute.org/GENE-E/) was used to cluster *r* heat maps.

### Supplementary Information

Supplementary Information.

## Data Availability

The data set used for analyses is available upon request from corresponding author.

## Abbreviations

*A1*: *APOBEC1*
*A2*: *APOBEC2*
*A3A*: *APOBEC3A*
*A3B*: *APOBEC3B*
*A3C*: *APOBEC3C*
*A3D*: *APOBEC3D*
*A3F*: *APOBEC3F*
*A3G*: *APOBEC3G*
*A3H*: *APOBEC3H*
*A4*: *APOBEC4*
ACC: Adrenocortical carcinoma
ADAR: Adenosine deaminase acting on RNA
AID: Activation-induced deaminase
APOBEC: Apolipoprotein B mRNA-editing enzyme catalytic polypeptide
BER: Base excision repair
BLCA: Bladder carcinoma
BRCA: Breast carcinoma
CESC: Cervical/endocervical cancer
CHOL: Cholangiocarcinoma
COADREAD: Colorectal adenocarcinoma
DBS: Double-base substitution
DLBC: Diffuse large B-cell lymphoma
DNA-Rep: DNA replication pathway
DRR: DNA repair/replication
eID: Exome indel
eSBS: Exome single-base substitution
ESCA: Esophageal carcinoma
FATLS: Fanconi anemia-translesion synthesis
gDBS: Genome double-base substitution
gID: Genome indel
gSBS: Genome single-base substitution
GBM: Glioblastoma multiforme
HNSC: Head and neck squamous cell carcinoma
HR: Homologous recombination
ID: Insertion/deletion; indel
KICH: Kidney chromophobe
KIRC: Kidney clear cell carcinoma
KIRP: Kidney papillary cell carcinoma
LAML: Acute myeloid leukemia
LGG: Low-grade glioma
LIHC: Hepatocellular carcinoma
LUAD: Lung adenocarcinoma
LUSC: Lung squamous cell carcinoma
MESO: Mesothelioma
MMR: Mismatch repair
NER: Nucleotide excision repair
NHEJ: Nonhomologous end-joining
OV: Ovarian serous cystadenocarcinoma
PAAD: Pancreatic adenocarcinoma
PCPG: Pheochromocytoma/paraganglioma
PRAD: Prostate adenocarcinoma
SARC: Sarcoma
SBS: Single-base substitution
SKCM: Skin melanoma
SNV: Single-nucleotide variant
STAD: Stomach adenocarcinoma
TGCT: Testicular germ cell tumor
THCA: Thyroid carcinoma
THYM: Thymoma
UCEC: Uterine corpus endometrial carcinoma
UCS: Uterine carcinosarcoma
UVM: Uveal melanoma
UV: Ultraviolet
