# Supplementary material for: The role of distinct APOBEC/ADAR mRNA levels in mutational signatures linked to aging and ultraviolet radiation
Source: Sci Rep. 2024 Jul 4;14:15395. doi: 10.1038/s41598-024-64986-6 (PMC11224270; doi:10.1038/s41598-024-64986-6)
Supplement: Supplementary file 1 — Supplementary Information. [file 41598_2024_64986_MOESM1_ESM.docx]

**The Role of Distinct *APOBEC/ADAR* mRNA Levels in Mutational Signatures
Linked to Aging and Ultraviolet Radiation - Supplementary Information**

Ahmadreza Niavarani^1*^

^1^ Digestive Oncology Research Center, Digestive Disease Research Institute, Tehran University of Medical Sciences, Tehran, Iran

^*^ Corresponding author; Tel: +98 21 8241 5290; FAX: +98 21 8241 5400; email: [arniavarani@gmail.com](mailto:arniavarani@gmail.com)

Supplementary Table S1. The number of *APOBEC/ADAR* mutations among individual TCGA cancers, as well as the ratio of total mutations per exome.

| **Cohort** | **Exomes** | **Total/exome** | **mut-*A1*** | **mut-*A2*** | **mut-*A3A*** | **mut-*A3B*** | **mut-*A3C*** | **mut-*A3D*** | **mut-*A3F*** | **mut-*A3G*** | **mut-*A3H*** | **mut-*A4*** | **mut-*AICDA*** | **mut-*ADAR*** | **mut-*ADARB1*** | **mut-*ADARB2*** |
| --- | --- | --- | --- | --- | --- | --- | --- | --- | --- | --- | --- | --- | --- | --- | --- | --- |
| PCPG | 184 | 0 | 0 | 0 | 0 | 0 | 0 | 0 | 0 | 0 | 0 | 0 | 0 | 0 | 0 | 0 |
| TGCT | 151 | 0 | 0 | 0 | 0 | 0 | 0 | 0 | 0 | 0 | 0 | 0 | 0 | 0 | 0 | 0 |
| THYM | 123 | 0 | 0 | 0 | 0 | 0 | 0 | 0 | 0 | 0 | 0 | 0 | 0 | 0 | 0 | 0 |
| UVM | 80 | 0 | 0 | 0 | 0 | 0 | 0 | 0 | 0 | 0 | 0 | 0 | 0 | 0 | 0 | 0 |
| CHOL | 36 | 0.056 | 0 | 0 | 0 | 0 | 0 | 1 | 0 | 0 | 0 | 1 | 0 | 0 | 0 | 0 |
| MESO | 82 | 0.024 | 1 | 0 | 0 | 0 | 0 | 0 | 0 | 0 | 0 | 0 | 0 | 0 | 1 | 0 |
| KICH | 66 | 0.045 | 0 | 0 | 0 | 0 | 0 | 0 | 0 | 1 | 0 | 0 | 2 | 0 | 0 | 0 |
| ACC | 92 | 0.033 | 0 | 0 | 0 | 0 | 1 | 0 | 0 | 0 | 0 | 0 | 0 | 0 | 2 | 0 |
| DLBC | 37 | 0.081 | 0 | 0 | 0 | 0 | 0 | 1 | 0 | 0 | 0 | 0 | 0 | 0 | 0 | 2 |
| LAML | 141 | 0.028 | 0 | 0 | 0 | 0 | 1 | 0 | 0 | 1 | 0 | 0 | 0 | 1 | 1 | 0 |
| UCS | 57 | 0.088 | 2 | 0 | 0 | 0 | 1 | 0 | 1 | 0 | 0 | 0 | 0 | 0 | 0 | 1 |
| SARC | 239 | 0.033 | 0 | 0 | 0 | 1 | 0 | 0 | 0 | 1 | 0 | 0 | 1 | 1 | 0 | 4 |
| THCA | 500 | 0.018 | 0 | 1 | 0 | 0 | 0 | 1 | 1 | 1 | 0 | 0 | 0 | 1 | 3 | 1 |
| PAAD | 178 | 0.056 | 0 | 0 | 0 | 1 | 0 | 1 | 0 | 1 | 1 | 0 | 0 | 2 | 1 | 3 |
| KIRC | 370 | 0.03 | 1 | 0 | 0 | 2 | 0 | 0 | 0 | 0 | 0 | 2 | 1 | 4 | 0 | 1 |
| PRAD | 498 | 0.032 | 3 | 1 | 0 | 4 | 0 | 1 | 1 | 0 | 0 | 0 | 1 | 1 | 1 | 3 |
| KIRP | 282 | 0.057 | 2 | 0 | 0 | 3 | 0 | 0 | 1 | 0 | 0 | 0 | 0 | 3 | 4 | 3 |
| LGG | 526 | 0.038 | 2 | 0 | 0 | 1 | 0 | 1 | 2 | 2 | 2 | 1 | 2 | 1 | 2 | 4 |
| ESCA | 185 | 0.108 | 2 | 1 | 0 | 0 | 0 | 1 | 0 | 3 | 1 | 0 | 3 | 4 | 2 | 3 |
| OV | 412 | 0.056 | 2 | 0 | 1 | 1 | 0 | 2 | 2 | 1 | 0 | 1 | 2 | 7 | 3 | 1 |
| LIHC | 365 | 0.077 | 0 | 1 | 1 | 1 | 1 | 0 | 2 | 1 | 1 | 3 | 3 | 3 | 4 | 7 |
| GBM | 403 | 0.084 | 2 | 0 | 1 | 2 | 2 | 1 | 3 | 3 | 3 | 1 | 5 | 2 | 2 | 7 |
| HNSC | 509 | 0.067 | 1 | 0 | 0 | 5 | 1 | 4 | 0 | 1 | 1 | 0 | 2 | 6 | 2 | 11 |
| CESC | 291 | 0.134 | 3 | 1 | 0 | 3 | 4 | 0 | 3 | 8 | 1 | 3 | 2 | 6 | 3 | 2 |
| BRCA | 1026 | 0.038 | 3 | 2 | 2 | 7 | 1 | 0 | 2 | 4 | 0 | 1 | 3 | 4 | 4 | 6 |
| BLCA | 411 | 0.139 | 2 | 2 | 1 | 4 | 3 | 3 | 2 | 2 | 3 | 7 | 2 | 14 | 3 | 9 |
| LUAD | 569 | 0.141 | 2 | 1 | 4 | 3 | 2 | 7 | 0 | 5 | 4 | 7 | 5 | 9 | 15 | 16 |
| STAD | 439 | 0.182 | 7 | 3 | 1 | 7 | 4 | 4 | 3 | 3 | 1 | 2 | 4 | 16 | 14 | 11 |
| LUSC | 485 | 0.179 | 12 | 2 | 2 | 6 | 2 | 4 | 7 | 3 | 2 | 3 | 10 | 12 | 5 | 17 |
| COADREAD | 559 | 0.279 | 11 | 7 | 7 | 8 | 7 | 9 | 6 | 13 | 4 | 11 | 10 | 18 | 24 | 21 |
| SKCM | 468 | 0.357 | 9 | 9 | 11 | 12 | 6 | 12 | 14 | 9 | 5 | 4 | 8 | 19 | 21 | 28 |
| UCEC | 531 | 0.616 | 22 | 20 | 9 | 24 | 11 | 26 | 25 | 16 | 12 | 15 | 24 | 43 | 43 | 37 |

Supplementary Table S2. The median and mean number of single-nucleotide (SNV) and indel variants in exomes with wild-type versus mutant *APOBEC/ADAR* genes.

| Gene mutation | Wild-type/ Mutant | Median SNV | | Median Indel | | Mean SNV | | | | Mean Indel | | | |
| --- | --- | --- | --- | --- | --- | --- | --- | --- | --- | --- | --- | --- | --- |
|  |  | Wild-type | Mutant | Wild-type | Mutant | Wild-type | Mutant | *P** | FDR | Wild-type | Mutant | *P** | FDR |
| *APOBEC1* | 10185/89 | 88 | 1286 | 4 | 20 | 284.2 ±10 | 5200.3 ±894.5 | 3.7e-07 | 5.24e-06 | 15.3 ±0.7 | 182.1 ±54.4 | 2.9e-03 | 4.0e-02 |
| *APOBEC2* | 10226/51 | 88 | 2402 | 4 | 30 | 286.9 ±9.9 | 7327.4 ±1509.6 | 2.3e-05 | 3.29e-04 | 15.9 ±0.8 | 156.7 ±36.4 | 3.1e-04 | 4.4e-03 |
| *APOBEC3A* | 10240/40 | 89 | 1264 | 4 | 16 | 315.3 ±13.2 | 3847.3 ±850 | 1.7e-04 | 2.40e-03 | 16.6 ±0.8 | 65.5 ±18.1 | 1.0e-02 | 1.4e-01 |
| *APOBEC3B* | 10181/95 | 88 | 1188 | 4 | 20 | 290.4 ±10.9 | 4467.7 ±827.6 | 2.2e-06 | 3.06e-05 | 15.3 ±0.7 | 176.3 ±41.9 | 2.2e-04 | 3.1e-03 |
| *APOBEC3C* | 10237/47 | 89 | 1573 | 4 | 34 | 311.6 ±12.6 | 4810 ±1163.8 | 3.5e-04 | 4.85e-03 | 16.3 ±0.8 | 130 ±31.9 | 8.7e-04 | 1.2e-02 |
| *APOBEC3D* | 10176/79 | 88 | 1549 | 4 | 21 | 274.1 ±10.5 | 5349.4 ±854.1 | 7.5e-08 | 1.04e-06 | 15.4 ±0.8 | 142.9 ±27.2 | 1.1e-05 | 1.6e-04 |
| *APOBEC3F* | 10184/75 | 88 | 2065 | 4 | 32 | 264.5 ±8.2 | 6321.6 ±827 | 2.4e-10 | 3.40e-09 | 15.6 ±0.8 | 129.9 ±26.4 | 4.7e-05 | 6.6e-04 |
| *APOBEC3G* | 10196/79 | 88 | 1596 | 4 | 14 | 292.8 ±11.1 | 4397.3 ±705.4 | 1.3e-07 | 1.75e-06 | 15.9 ±0.8 | 106.5 ±22.3 | 1.2e-04 | 1.6e-03 |
| *APOBEC3H* | 10244/41 | 89 | 1144 | 4 | 30 | 303.1 ±11.8 | 6845.3 ±1496.8 | 8.6e-05 | 1.20e-03 | 16.4 ±0.8 | 123.9 ±29.9 | 8.8e-04 | 1.2e-02 |
| *APOBEC4* | 10219/62 | 88 | 1059 | 4 | 22 | 291.8 ±11.6 | 5383 ±910.4 | 5.6e-07 | 7.84e-06 | 15.9 ±0.8 | 128.1 ±32.5 | 1.0e-03 | 1.4e-02 |
| *AICDA* | 10179/90 | 88 | 937 | 4 | 14 | 282.8 ±10 | 5351.6 ±960.1 | 9.1e-07 | 1.27e-05 | 15.8 ±0.8 | 93.4 ±19.5 | 1.4e-04 | 1.9e-03 |
| *ADAR* | 10066/177 | 87 | 984 | 4 | 18 | 254.2 ±8.2 | 4049.1 ±558.1 | 1.6e-10 | 2.21e-09 | 14.2 ±0.8 | 152 ±19 | 1.3e-11 | 1.8e-10 |
| *ADARB1* | 10085/160 | 87 | 1225 | 4 | 27 | 248.7 ±7.5 | 4686.8 ±548.4 | 1.4e-13 | 1.99e-12 | 13.8 ±0.6 | 157.1 ±20.7 | 9.6e-11 | 1.3e-09 |
| *ADARB2* | 10022/198 | 87 | 504 | 4 | 12 | 250.4 ±7.5 | 3482.3 ±494.5 | 5.3e-10 | 7.45e-09 | 14.7 ±0.8 | 105.2 ±16.8 | 2.0e-07 | 2.8e-06 |

* Unpaired two-tailed Student’s t test.

Supplementary Table S3. The mean number of single-nucleotide variant (SNV) and indel (Ind) variant classes among exomes with synonymous versus nonsynonymous *APOBEC/ADAR* mutations. Those exomes with nonsynonymous *A3C, AICDA,* and *ADARB1* mutations were enriched in T>N and/or C>N variants compared to exomes with synonymous ones, and those exomes with any nonsynonymous *A1, A3B, A3C, A3H*, and *ADAR* mutations were enriched in Ind-T and/or Ind-C variants.

| **Gene mutation** | **Synonymous/ Non-synonymous** | **T>N** | | | | **C>N** | | | | **Ind-T** | | | | **Ind-C** | | | |
| --- | --- | --- | --- | --- | --- | --- | --- | --- | --- | --- | --- | --- | --- | --- | --- | --- | --- |
|  |  | **Synonymous** | **Non-synonymous** | ***P**** | **FDR** | **Synonymous** | **Non-synonymous** | ***P**** | **FDR** | **Synonymous** | **Non-synonymous** | ***P**** | **FDR** | **Synonymous** | **Non-synonymous** | ***P**** | **FDR** |
| *APOBEC1* | 21/89 | 441 ±272.5 | 1029.4 ±181.6 | 8.0e-02 | NS | 2884.7 ±1601.7 | 4171 ±787.5 | 4.8e-01 | NS | 18.6 ±9.9 | 111.8 ±29 | 3.0e-03 | 4.2e-02 | 11.4 ±6 | 70.3 ±26.3 | 3.1e-02 | NS |
| *APOBEC2* | 18/51 | 1063.7 ±330.1 | 1295.7 ±233.8 | 5.7e-01 | NS | 5630.6 ±1650.5 | 6031.7 ±1361.3 | 8.5e-01 | NS | 129.9 ±66.7 | 105.9 ±23.5 | 7.4e-01 | NS | 18.1 ±6.7 | 50.9 ±15.4 | 5.5e-02 | NS |
| *APOBEC3A* | 15/40 | 549.1 ±346.1 | 879.3 ±254.2 | 4.5e-01 | NS | 2483.1 ±1394.2 | 2968.1 ±609.8 | 7.5e-01 | NS | 31.9 ±18 | 42.3 ±12.2 | 6.4e-01 | NS | 21.4 ±13.4 | 23.2 ±7.3 | 9.1e-01 | NS |
| *APOBEC3B* | 19/94 | 498.4 ±229.3 | 794.1 ±159.1 | 3.0e-01 | NS | 1950.5 ±688.4 | 3673.5 ±736 | 9.2e-02 | NS | 22.7 ±7.7 | 113.7 ±25.1 | 7.7e-04 | 1.1e-02 | 12.2 ±4.6 | 62.7 ±17.7 | 6.7e-03 | NS |
| *APOBEC3C* | 11/47 | 202.8 ±166.4 | 767.1 ±186.9 | 3.0e-02 | NS | 851 ±500.3 | 4042.8 ±987.1 | 5.6e-03 | NS | 4.1 ±1.7 | 91 ±25.8 | 1.6e-03 | 2.2e-02 | 2.3 ±0.6 | 39 ±8.4 | 7.6e-05 | 1.1e-03 |
| *APOBEC3D* | 40/79 | 1143.6 ±267.4 | 985.8 ±172.2 | 6.2e-01 | NS | 4261.8 ±785.9 | 4363.5 ±703.2 | 9.2e-01 | NS | 89.5 ±22.6 | 94.8 ±17.1 | 8.5e-01 | NS | 41.3 ±11.6 | 48.1 ±11.2 | 6.8e-01 | NS |
| *APOBEC3F* | 36/75 | 989.6 ±311.8 | 1200.3 ±182.3 | 5.6e-01 | NS | 6227.7 ±1887.7 | 5121.3 ±668.5 | 5.8e-01 | NS | 90.2 ±32.7 | 92.2 ±19.8 | 9.6e-01 | NS | 28.1 ±14.5 | 37.7 ±9 | 5.7e-01 | NS |
| *APOBEC3G* | 20/79 | 480.2 ±278.7 | 813.7 ±158 | 3.1e-01 | NS | 4280.2 ±2674.9 | 3583.5 ±563 | 8.0e-01 | NS | 70.8 ±48.2 | 77.6 ±17.1 | 8.9e-01 | NS | 53.1 ±31.5 | 28.8 ±6.5 | 4.6e-01 | NS |
| *APOBEC3H* | 10/41 | 702.3 ±332.8 | 1332.3 ±313.1 | 1.8e-01 | NS | 3529.4 ±1799.3 | 5513 ±1214 | 3.7e-01 | NS | 25.7 ±11.7 | 91.9 ±23.3 | 1.4e-02 | NS | 10.8 ±7.3 | 32 ±8.9 | 7.3e-02 | NS |
| *APOBEC4* | 14/62 | 1805.2 ±590.3 | 1133.8 ±208.4 | 3.0e-01 | NS | 6175 ±1667.4 | 4249.2 ±726.2 | 3.0e-01 | NS | 112.1 ±35.1 | 85.9 ±20.8 | 5.3e-01 | NS | 44.1 ±19.5 | 42.2 ±14 | 9.4e-01 | NS |
| *AICDA* | 26/90 | 371.4 ±138.2 | 867.2 ±148.6 | 1.7e-02 | NS | 2216.5 ±550.9 | 4484.4 ±850.6 | 2.7e-02 | NS | 98.8 ±38.5 | 67.2 ±13.9 | 4.4e-01 | NS | 50.5 ±21.9 | 26.2 ±6.7 | 3.0e-01 | NS |
| *ADAR* | 52/177 | 558.6 ±145.6 | 712.1 ±98.8 | 3.8e-01 | NS | 2371.7 ±532.2 | 3337 ±487.1 | 1.8e-01 | NS | 44.3 ±9.9 | 100.7 ±12.5 | 5.1e-04 | 7.1e-03 | 23.9 ±5.3 | 51.2 ±7.4 | 3.1e-03 | 4.4e-02 |
| *ADARB1* | 50/151 | 346.3 ±92.2 | 967.5 ±126.3 | 1.0e-04 | 1.4e-03 | 3053.7 ±1158.5 | 3719.3 ±442.6 | 5.9e-01 | NS | 105 ±44.9 | 103.6 ±13.9 | 9.8e-01 | NS | 78.5 ±43.9 | 53.4 ±7.8 | 5.8e-01 | NS |
| *ADARB2* | 75/198 | 504.9 ±121.9 | 645.3 ±100.3 | 3.8e-01 | NS | 2541.8 ±593.8 | 2837 ±423.1 | 6.9e-01 | NS | 39.5 ±12.4 | 70.8 ±11.7 | 6.8e-02 | NS | 27.3 ±9.7 | 34.3 ±5.9 | 5.4e-01 | NS |

* Unpaired two-tailed Student’s t test.

FDR: *Benjamini–*Hochberg false discovery rate.

NS: statistically not significant.

Supplementary Table S4. The rotated component matrix resulted from principal component (PC) analysis of the covariates potentially implicated in mutations across exomes/genomes. Varimax rotation with Kaiser normalization was used in order to extract the components. The cut-off for the correlation between the covariate and the estimated component was considered to be 0.4, shown in bold.

| No. | Component | 1 | 2 | 3 | 4 | 5 | 6 | 7 | 8 |
| --- | --- | --- | --- | --- | --- | --- | --- | --- | --- |
| 1 | Age | .048 | .058 | .067 | .033 | **.703** | .007 | .057 | -.001 |
| 2 | UV | .095 | .080 | **.768** | -.029 | -.069 | -.007 | -.038 | -.078 |
| 3 | mutBER | **.900** | .038 | -.004 | .004 | .005 | .037 | .026 | .030 |
| 4 | mutNER | **.939** | .027 | -.012 | .006 | -.001 | .047 | .003 | .035 |
| 5 | mutMMR | **.916** | .019 | -.030 | .011 | .000 | .027 | .001 | .039 |
| 6 | mutHR | **.921** | .033 | -.023 | .024 | .014 | .056 | -.002 | .034 |
| 7 | mutNHEJ | **.807** | .031 | .012 | .000 | .020 | .066 | .019 | .018 |
| 8 | mutFATLS | **.937** | .036 | -.002 | .020 | .011 | .050 | -.003 | .045 |
| 9 | mutRepl | **.930** | .022 | -.008 | .007 | .010 | .043 | .000 | .021 |
| 10 | *A1*-mRNA | .004 | -.055 | -.069 | -.114 | **.575** | .000 | .009 | -.161 |
| 11 | *A2*-mRNA | -.025 | .189 | -.184 | -.186 | -.065 | .062 | .126 | -.480 |
| 12 | *A3A*-mRNA | .006 | .024 | -.063 | **.775** | -.059 | -.002 | .038 | -.074 |
| 13 | *A3B*-mRNA | .037 | .288 | -.031 | **.689** | .076 | .011 | .000 | .045 |
| 14 | *A3C*-mRNA | .017 | **.634** | **.464** | .236 | .083 | .016 | .020 | -.035 |
| 15 | *A3D*-mRNA | .024 | **.852** | .117 | .100 | .071 | .003 | -.008 | .046 |
| 16 | *A3F*-mRNA | .008 | **.822** | .092 | .072 | .045 | .023 | .003 | .098 |
| 17 | *A3G*-mRNA | .025 | **.855** | .078 | .072 | -.010 | -.025 | -.009 | -.015 |
| 18 | *A3H*-mRNA | .020 | **.765** | -.053 | -.046 | -.046 | -.027 | -.013 | .005 |
| 19 | *A4-*mRNA | -.020 | .070 | -.173 | -.179 | .043 | .136 | .160 | **.552** |
| 20 | *AICDA*-mRNA | -.007 | .081 | -.001 | -.036 | -.058 | -.086 | -.077 | **.405** |
| 21 | *ADAR-*mRNA | -.024 | .190 | **.406** | .167 | .130 | .038 | .053 | .340 |
| 22 | *ADARB1*-mRNA | -.049 | .129 | **.715** | -.166 | -.099 | .015 | .022 | .013 |
| 23 | *ADARB2*-mRNA | -.051 | -.078 | .105 | -.107 | -.529 | .002 | .071 | -.216 |
| 24 | mut*A1* | .385 | -.007 | .005 | -.007 | -.033 | -.050 | .389 | -.048 |
| 25 | mut*A2* | .367 | .006 | -.003 | -.024 | .000 | .213 | -.206 | -.071 |
| 26 | mut*A3A* | .218 | -.002 | .046 | -.027 | .010 | -.125 | -.535 | .014 |
| 27 | mut*A3B* | .332 | -.023 | .081 | .007 | .081 | -.125 | **.508** | -.105 |
| 28 | mut*A3C* | .331 | .011 | -.052 | -.050 | -.064 | -.615 | -.127 | .139 |
| 29 | mut*A3D* | .331 | .009 | .016 | -.023 | .046 | **.430** | -.068 | -.022 |
| 30 | mut*A3F* | **.423** | -.010 | .025 | -.013 | -.017 | .183 | -.167 | -.113 |
| 31 | mut*A3G* | .293 | -.006 | .010 | -.005 | -.004 | **.419** | -.037 | -.004 |
| 32 | mut*A3H* | .299 | -.021 | -.022 | .011 | -.110 | .244 | .227 | .202 |
| 33 | mut*A4* | .327 | -.001 | -.037 | -.002 | -.032 | .259 | -.242 | -.021 |
| 34 | mut*AICDA* | .306 | -.010 | -.046 | -.016 | -.100 | .338 | .034 | .207 |
| 35 | mut*ADAR* | **.427** | .022 | .035 | -.002 | .091 | -.036 | .100 | -.064 |
| 36 | mut*ADARB1* | **.462** | -.026 | .028 | -.005 | .055 | .032 | -.307 | -.050 |
| 37 | mut*ADARB2* | .358 | -.030 | .038 | .036 | .012 | .059 | .080 | .062 |

Supplementary Table S5. The rotated component matrix resulted from principal component (PC) analysis of the covariates potentially implicated in mutations across cancer types. Varimax rotation with Kaiser Normalization was used in order to extract the components. The cut-off for the correlation between the covariate and the estimated component was considered to be 0.4, as shown in bold.

| No. | Component | 1 | 2 | 3 | 4 | 5 |
| --- | --- | --- | --- | --- | --- | --- |
| 1 | Age | .268 | .103 | -.006 | -.029 | **.642** |
| 2 | UV | .168 | .065 | **.915** | .024 | -.038 |
| 3 | mutBER | **.981** | .009 | .008 | .099 | .113 |
| 4 | mutNER | **.982** | .029 | -.013 | .087 | .089 |
| 5 | mutMMR | **.983** | -.023 | -.053 | .063 | .092 |
| 6 | mutHR | **.967** | .021 | -.034 | .134 | .137 |
| 7 | mutNHEJ | **.958** | .009 | .044 | .151 | .169 |
| 8 | mutFATLS | **.968** | .104 | -.003 | .138 | .125 |
| 9 | mutRepl | **.982** | -.007 | -.007 | .101 | .128 |
| 10 | *A1*-mRNA | .113 | -.169 | -.204 | -.120 | **.501** |
| 11 | *A2*-mRNA | -.138 | .211 | -.179 | -.332 | .004 |
| 12 | *A3A*-mRNA | .022 | .061 | -.147 | **.841** | -.063 |
| 13 | *A3B*-mRNA | .253 | .178 | -.184 | **.861** | .094 |
| 14 | *A3C*-mRNA | .011 | **.517** | **.641** | .363 | .145 |
| 15 | *A3D*-mRNA | -.050 | **.918** | .133 | .118 | .049 |
| 16 | *A3F*-mRNA | .119 | **.775** | .188 | -.023 | .234 |
| 17 | *A3G*-mRNA | -.041 | **.936** | .083 | .062 | .029 |
| 18 | *A3H*-mRNA | -.013 | **.954** | -.037 | -.040 | -.072 |
| 19 | *A4-*mRNA | **.799** | .010 | -.191 | -.168 | .029 |
| 20 | *AICDA*-mRNA | -.045 | **.742** | -.166 | -.047 | -.235 |
| 21 | *ADAR-*mRNA | .063 | .036 | .147 | .131 | **.735** |
| 22 | *ADARB1*-mRNA | -.135 | .094 | **.804** | -.190 | .096 |
| 23 | *ADARB2*-mRNA | -.157 | -.128 | **.513** | -.235 | -.239 |
| 24 | mut*A1* | **.803** | -.146 | -.086 | -.035 | -.013 |
| 25 | mut*A2* | **.970** | .001 | .067 | -.020 | .063 |
| 26 | mut*A3A* | **.832** | .015 | .317 | -.007 | .163 |
| 27 | mut*A3B* | **.915** | -.007 | .104 | .122 | .111 |
| 28 | mut*A3C* | **.763** | -.091 | -.110 | .194 | -.106 |
| 29 | mut*A3D* | **.767** | .374 | -.093 | -.045 | .192 |
| 30 | mut*A3F* | **.935** | -.052 | .093 | -.013 | -.078 |
| 31 | mut*A3G* | **.733** | -.178 | -.074 | .339 | .020 |
| 32 | mut*A3H* | **.911** | -.043 | .009 | .034 | .122 |
| 33 | mut*A4* | **.654** | .090 | -.122 | .097 | .366 |
| 34 | mut*AICDA* | **.793** | -.192 | -.131 | .081 | -.009 |
| 35 | mut*ADAR* | **.919** | .007 | -.005 | .166 | .215 |
| 36 | mut*ADARB1* | **.927** | -.047 | .050 | -.015 | .126 |
| 37 | mut*ADARB2* | **.817** | .302 | -.019 | .055 | .078 |


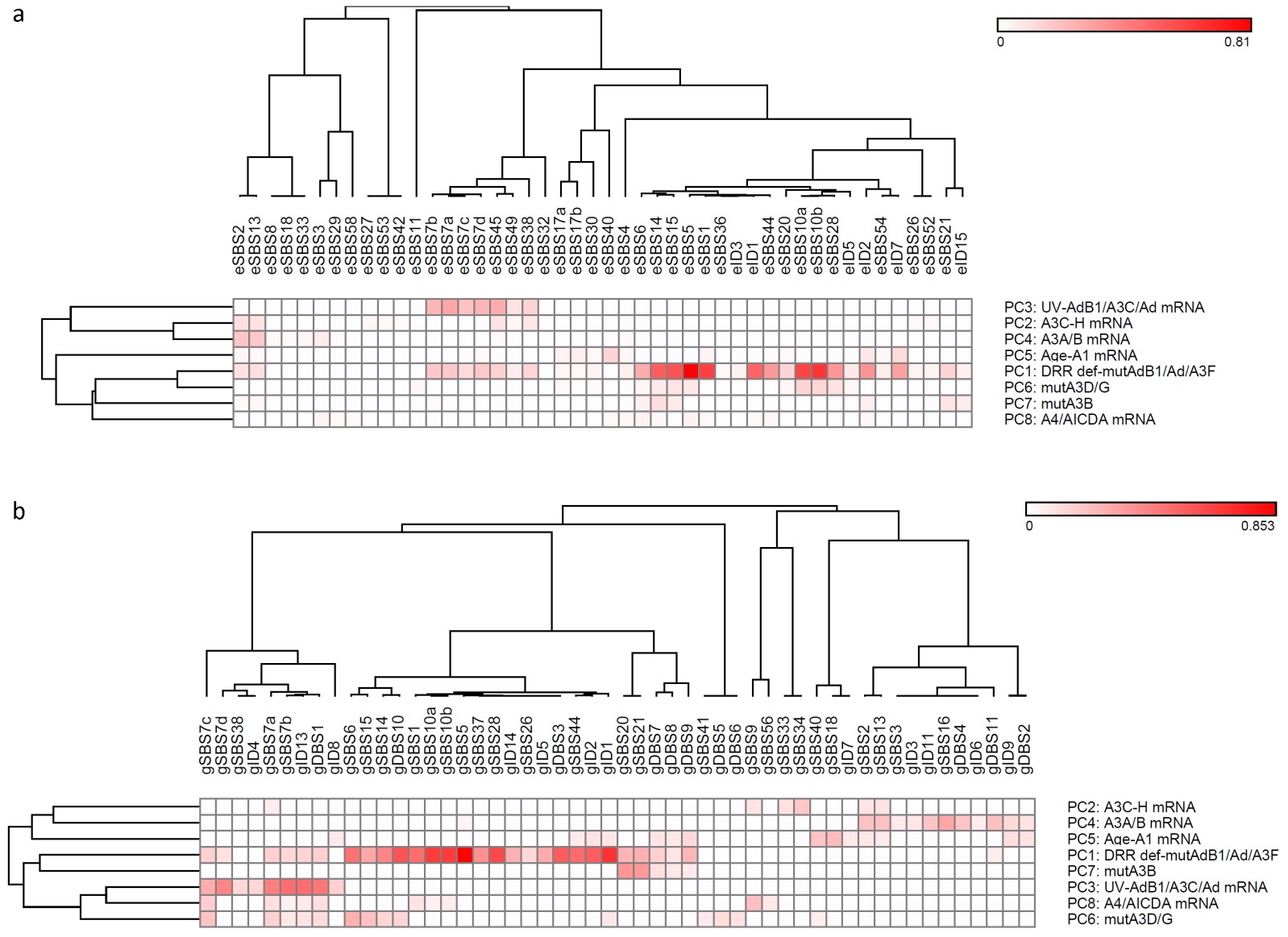


Supplementary Figure S1. Clustering analyses of the correlation (*r* coefficient) between pancancer signatures and principal components potentially contributing to positive impact on cancer mutagenesis. (a) Pancancer exome signatures. (b) Pancancer genome signatures. While the dominant components, including DRR defects, UV-*ADARB1,* and *A3A/B* mRNA levels (in genome analysis), generally shaped the clusters, the less prominent ones like age-*A1* mRNA level, mut*A3B*, and mut*A3D/G* made small clusters or subclusters.

Abbreviations: A1, *APOBEC1*; A3, *APOBEC3*; Ad, *ADAR*; AdB1, *ADARB1*.


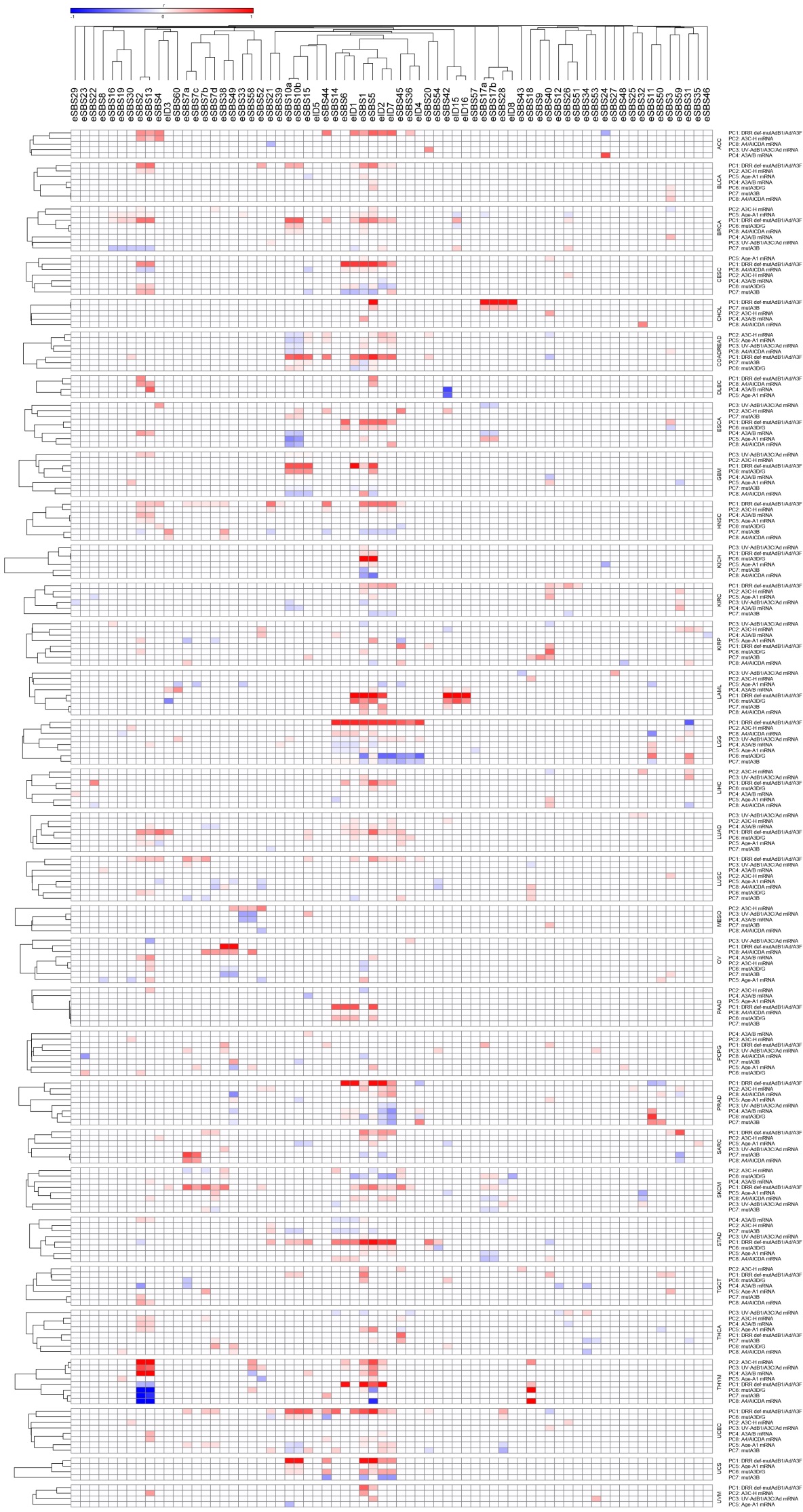


Supplementary Figure S2. Detailed correlation of the exome signatures with principal components (PCs) potentially implicated in cancer mutagenesis in individual cancers, as stratified by cancers and clustered using Pearson regression *r* coefficient.

Abbreviations: A1, *APOBEC1*; A3, *APOBEC3*; Ad, *ADAR*; AdB1, *ADARB1*.


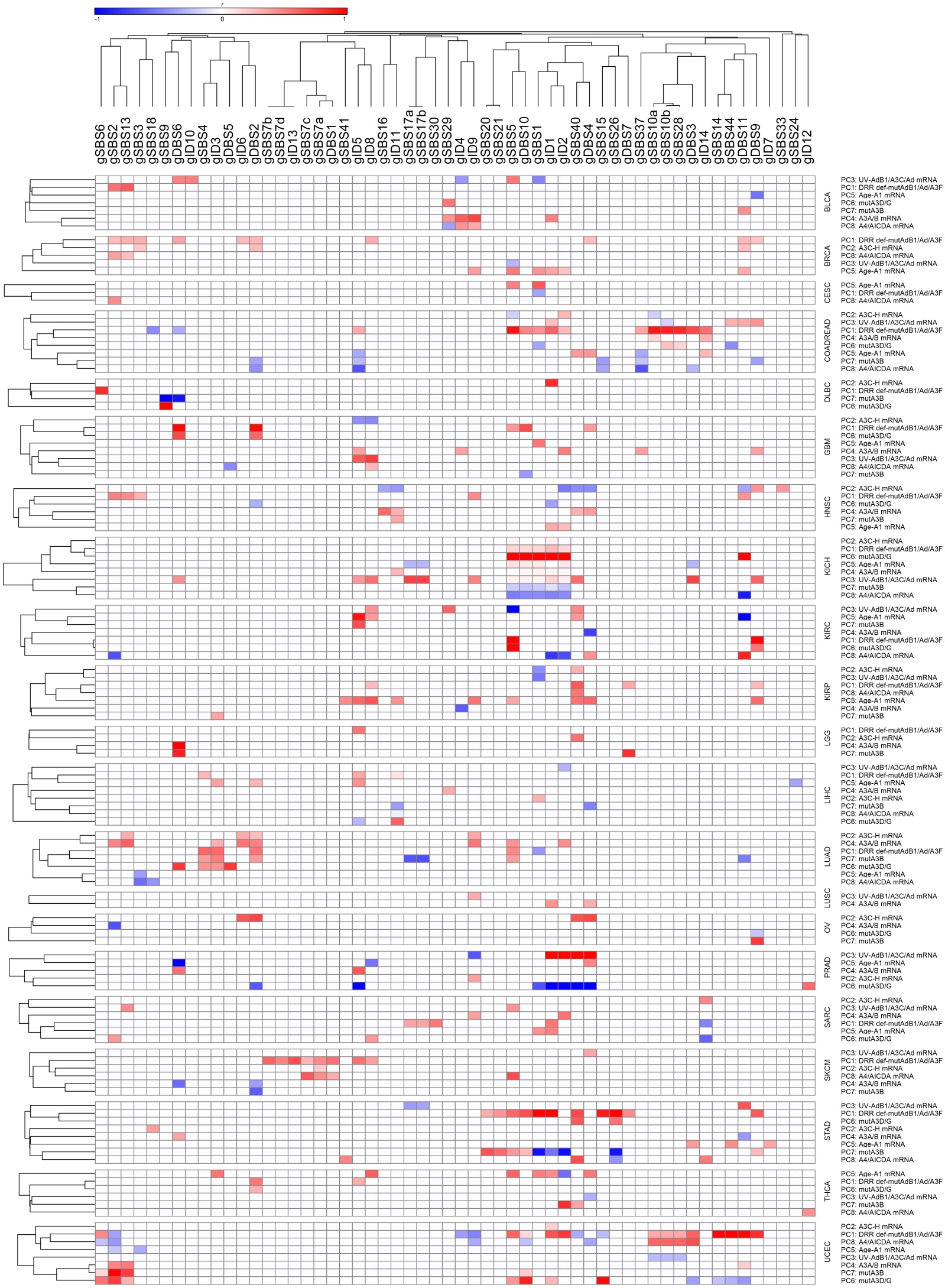


Supplementary Figure S3. Detailed correlation of the genome signatures with principal components (PCs) potentially implicated in cancer mutagenesis in individual cancers, as stratified by cancers and clustered using Pearson regression *r* coefficient.

Abbreviations: A1, *APOBEC1*; A3, *APOBEC3*; Ad, *ADAR*; AdB1, *ADARB1*.


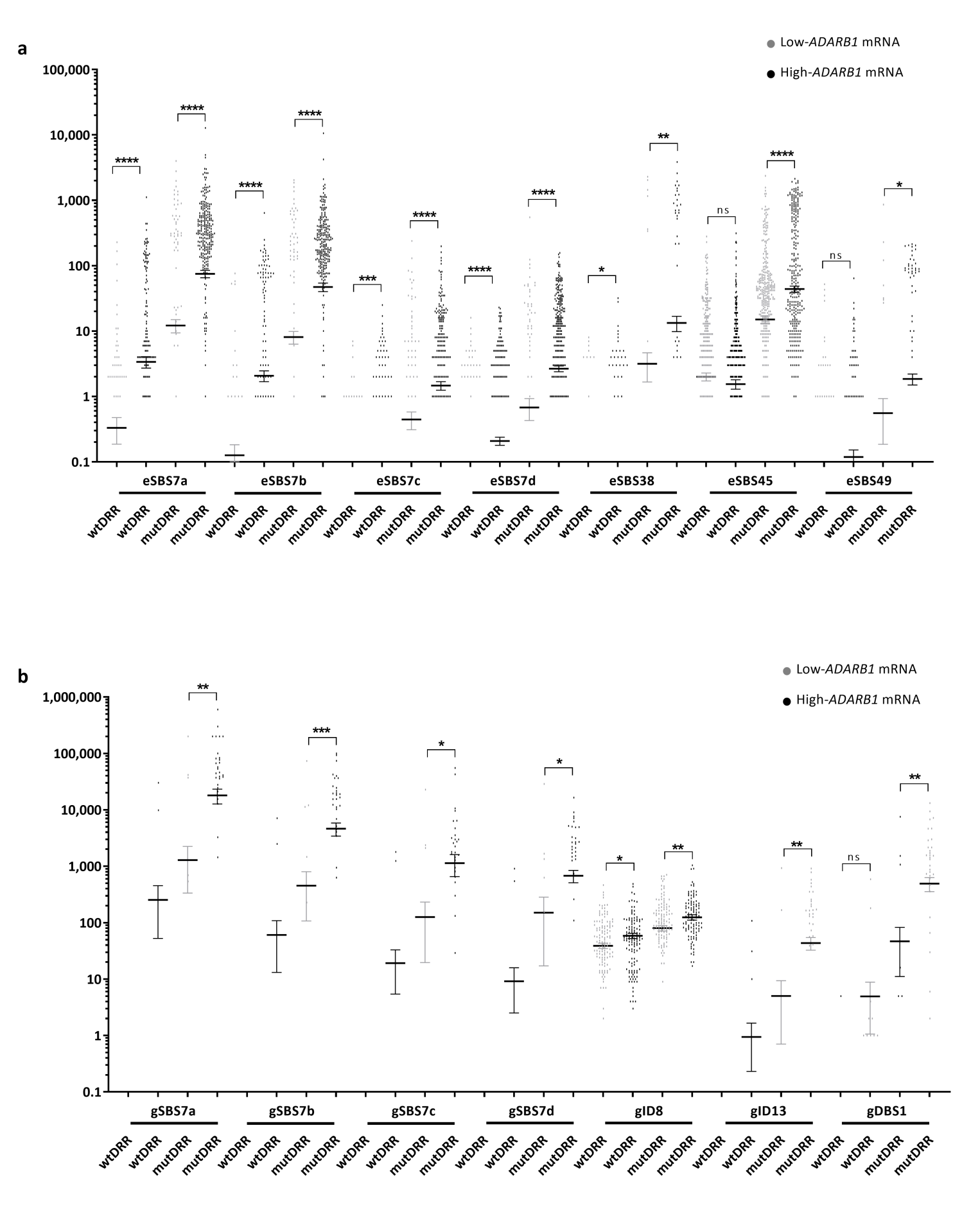


Supplementary Figure S4. The number of pancancer (a) exome and (b) genome mutational signatures correlated with *ADBARB1* mRNA level, as classified by DRR mutational status. There were generally enough samples in the mutDRR class, allowing fair comparison between low- and high-*ADARB1* mRNA level subclasses.

An unpaired two-tailed t-test with Welch's correction was used in order to test the statistical significance of low- vs. high-*ADARB1* mRNA level subclasses. wt, wild type; mut, mutated.

ns, not significant; *, *p* < 0.05; **, *p* < 0.01; ***, *p* < 0.001; ****, *p* < 0.0001.


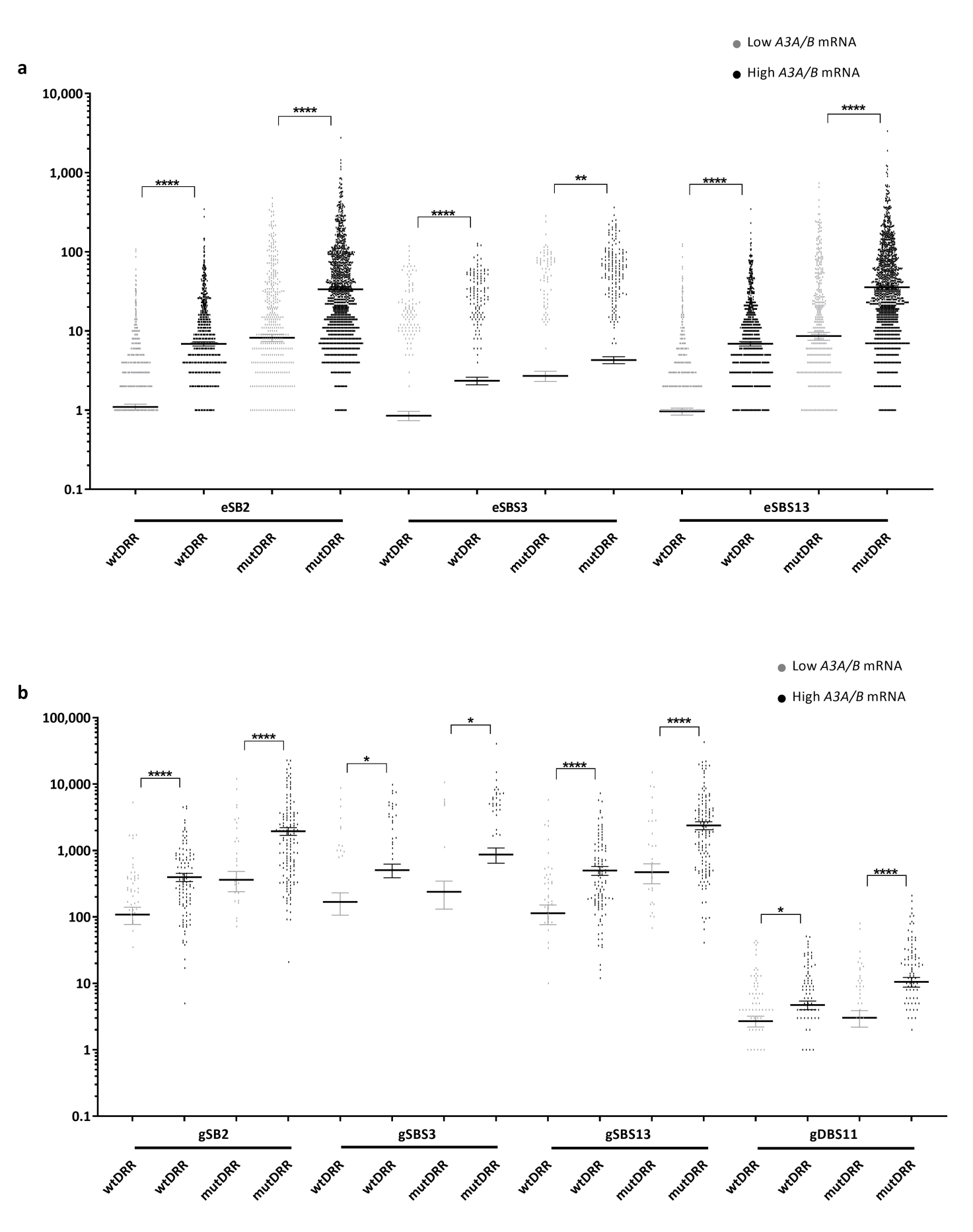


Supplementary Figure S5. The number of pancancer (a) exome and (b) genome mutational signatures correlated with *A3A/B* mRNA level, as classified by DRR mutational status. The number of all shown mutational signatures was significantly higher in the high-*A3A/B* mRNA level subclass across both wtDRR and mutDRR classes.

An unpaired two-tailed t-test with Welch's correction was used in order to test the statistical significance of low- vs. high-*A3A/B* mRNA level subclasses. wt, wild type; mut, mutated.

ns, not significant; *, *p* < 0.05; **, *p* < 0.01; ***, *p* < 0.001; ****, *p* < 0.0001.


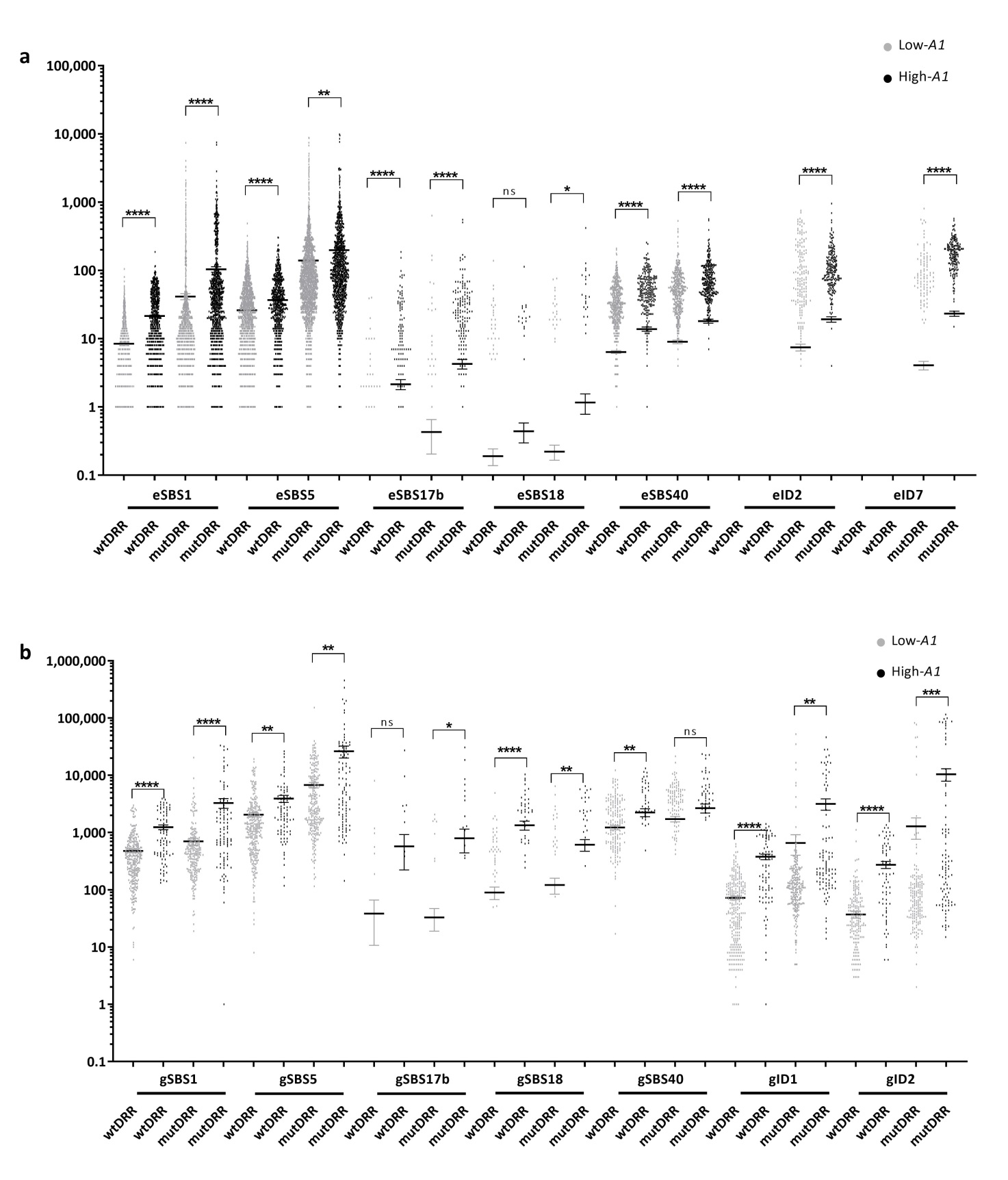


Supplementary Figure S6. The number of pancancer (a) exome and (b) genome mutational signatures correlated with the *A1* mRNA level, as classified by DRR mutational status. The number of shown mutational signatures was significantly higher in the high-*A1* mRNA level subclass across most wtDRR and mutDRR classes.

An unpaired two-tailed t-test with Welch's correction was used in order to test the statistical significance of low- vs. high-*A1* mRNA level subclasses. wt, wild type; mut, mutated.

ns, not significant; *, *p* < 0.05; **, *p* < 0.01; ***, *p* < 0.001; ****, *p* < 0.0001.


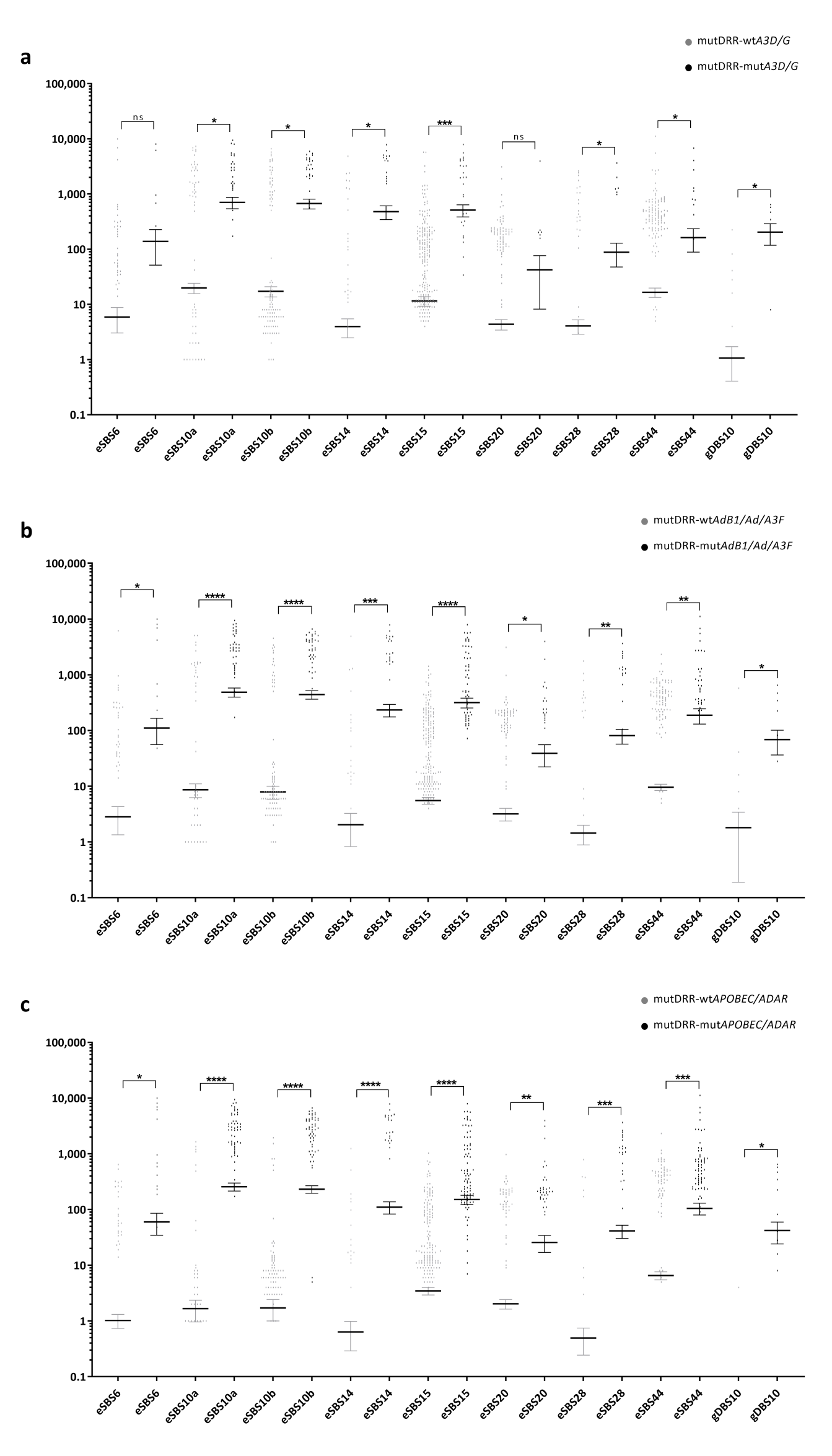


Supplementary Figure S7. The number of pancancer exome and genome mutational signatures correlated with (a) mut*A3D/G*, (b) mut*A3F/ADARB1/ADAR*, and (c) mut*APOBEC/ADAR*, all in the mutant DRR class. While most mutational signatures showed significantly discrepant when the mutational status of *A3D/G* was used for further classification, all mutational signatures showed significantly higher in the mut*A3F/ADARB1/ADAR* and mut*APOBEC/ADAR* subclasses.

An unpaired two-tailed t-test with Welch's correction was used in order to test the statistical significance of wild-type (wt) vs. mutated (mut) subclasses.

ns, not significant; *, *p* < 0.05; **, *p* < 0.01; ***, *p* < 0.001; ****, *p* < 0.0001.
